# The mechanism of secreted frizzled-related protein 1 in alleviating cardiomyocyte injury and heart failure

**DOI:** 10.3389/fcvm.2025.1676224

**Published:** 2026-01-16

**Authors:** Yu He, Tianrun Liu, Li Chen, Zimeng Ge, Xuefeng Chang, Deli Zou

**Affiliations:** 1 Clinical Medical College, Beihua University, Jilin, Jilin, China; 2 College of Basic Medicine, Jiamusi University, Jiamusi, Heilongjiang, China; 3 Beihua University Affiliated Hospital, Jilin City, China

**Keywords:** cardiomyocyte injury, damaged cardiomyocytes, HF, secreted frizzled-related protein 1, Wnt/β-catenin signaling pathway

## Abstract

Heart failure (HF) is a clinical syndrome characterized by impairment of the heart’s pumping function. Its core pathological basis is the vicious cycle of “injury reconstruction decompensation” triggered by cardiomyocyte damage. This review aims to systematically elucidate the molecular mechanism by which secreted frizzled-related protein 1 (Sfrp1; all proteins mentioned in the article are mouse genes, and Sfrp1 is used as the abbreviation) alleviates myocardial injury and delays the progression of HF through a multi-pathway interaction network. The main contents include (1) the core pathological mechanism of HF, such as oxidative stress (excessive ROS leads to calcium overload and mitochondrial apoptosis), autophagy disorder (the AngII/β5i axis inhibits protective autophagy), and abnormal apoptosis (imbalance of Bax/Bcl-2 triggers cardiomyocyte loss); (2) the structural features of Sfrp1, a secretory glycoprotein rich in cysteine domains (CRD), which inhibits the classical Wnt/β-catenin pathway by competitively binding to Wnt ligands; (3) the Sfrp1 six-layer protective mechanisms of Sfrp1: antagonizing the Wnt pathway to reduce ROS production and fibrosis; activating the Hippo/Notch pathways to inhibit pathological proliferation; promoting autophagy; downregulating Bax/Cyt c/Caspase-3; upregulating Bcl-2 to inhibit apoptosis; and improving calcium metabolism disorders by upregulating SERCA2a/MICU1. The conclusion suggests that coordinated regulation of these pathways by Sfrp1 interrupts the vicious cycle of HF. As a multi-target intervention molecule, Sfrp1 offers a novel approach to the targeted treatment of HF.

## 1 Introduction

Heart failure (HF) is a clinical syndrome characterized by impairment of the heart’s pumping function, with a high clinical incidence and poor prognosis, posing a significant burden on public health. Its core pathological basis lies in cardiomyocyte damage, in which the structure and function of cardiomyocytes are impaired due to ischemia, inflammation, infection, or toxins. Such damage not only directly leads to a decrease in the efficiency of the heart’s pumping function but also triggers a vicious cycle of “damage–remodeling–decompensation” (1). Damaged cardiomyocytes release specific biomarkers (such as cTnT/I, CK-MB, and LDH) and inflammatory factors (such as IL-6) into the circulation. Meanwhile, increased ventricular pressure/capacity load significantly elevates plasma BNP (brain natriuretic peptide) and ANP (atrial natriuretic peptide) levels. The latter has become a routine indicator for clinical diagnosis and assessment of HF (2, 3). Secreted frizzled-related protein 1 (Sfrp1), a secreted glycoprotein containing a cysteine-rich domain (CRD), regulates multiple signaling pathways by competitively binding Wnt ligands and has emerged as a potential target for intervention in the aforementioned pathological processes (4). This review systematically elaborates on the molecular mechanisms by which Sfrp1 alleviates myocardial injury and delays HF progression through a multi-pathway network, providing a theoretical basis for targeted HF therapy (4).

## 2 The core pathological mechanism network of HF

In the field of cardiovascular diseases, HF represents the final stage of the development of various heart disorders. Its pathogenesis is complex and is closely related to cardiomyocyte damage (5). When cells experience irreparable DNA damage—such as that caused by chemotherapy drugs or ionizing radiation—or receive developmental cues (like the separation of embryonic fingers and toes), apoptosis-related genes activate a “death program.” During this process, the cells shrink, the chromatin condenses toward the edges, and ultimately, the cell breaks down into intact apoptotic bodies. These bodies are efficiently cleared away by macrophages without any leakage of cellular contents, which helps prevent an inflammatory response. This “quiet death” occurs trillions of times each day within the human body, helping to maintain tissue stability and the proper function of organs.

Autophagy originally serves as a self-degradation mechanism for cells to maintain homeostasis. However, it becomes abnormal during the process of HF. Excessive autophagy or insufficient autophagy can both interfere with the normal metabolism and function of cardiac muscle cells, exacerbating cell damage. Oxidative stress occurs due to the imbalance between the body’s oxidation and antioxidant systems, resulting in the production of a large amount of reactive oxygen species (ROS), which attack lipids, proteins, and DNA within cardiac muscle cells, damaging the cell structure and inducing cell apoptosis (6, 7). During the process of myocardial fibrosis, fibroblasts become activated and secrete large amounts of extracellular matrix, replacing the damaged cardiomyocytes, thereby increasing the stiffness of the heart and reducing its compliance, which affects the pumping function of the heart (8). In adult individuals, the proliferative capacity of cardiomyocytes is already profoundly restricted. During HF, these cells fail to undergo effective proliferative repair in response to injury. Instead, they may exacerbate the burden on the heart due to abnormal compensatory reactions. These pathological physiological mechanisms are interrelated and mutually influential, collectively forming a complex pathological network of HF, which lays the foundation for further in-depth analysis of the pathological mechanism of HF.

### 2.1 The relationship between oxidative stress and cardiomyocyte damage

#### 2.1.1 Oxygen metabolism of cardiomyocytes under physiological conditions

The heart, as a highly oxygen-consuming organ, accounts for <5% of the body's total weight, yet it consumes 7% of the body's total oxygen. Under normal circumstances, fatty acid β-oxidation plays a major role in the energy supply of the myocardium (9, 10). Metabolomics analysis shows that fatty acid β-oxidation—a high oxygen-dependent metabolic pathway—requires more molecular oxygen flux for ATP synthesis than alternative pathways like glycolysis (11). Notably, fatty acid β-oxidation is a precursor step for myocardial energy production: It provides reducing equivalents (NADH/FADH2) and acetyl-coenzyme A (CoA) for oxidative phosphorylation, the core process of ATP generation in cardiomyocytes. Usually in a resting state, the myocardium prefers to utilize fatty acids because the high oxygen supply and abundant mitochondrial structure of the heart are suitable for the complete oxidation of fatty acids. Therefore, fatty acid β-oxidation serves as a precursor step to provide reducing equivalents (NADH/FADH2) and acetyl-CoA for oxidative phosphorylation, which is the final pathway for energy supply and directly generates ATP to maintain the continuous contraction of the myocardium.

#### 2.1.2 Under pathological conditions, excessive production of reactive oxygen species leads to an imbalance in the heart's antioxidant system

However, under conditions of redox homeostasis imbalance, electron leakage from substrate-overloaded mitochondrial electron transfer chain complexes in cardiomyocytes causes a dose-dependent increase in ROS generation (12). Moreover, this oxygen metabolic stress, along with the excessive production of ROS, may trigger a positive feedback loop that accelerates the depolarization process of the mitochondrial membrane potential in cardiac cells. This pathological process is closely related to arrhythmia and contractile dysfunction. For ROS, achieving this process only requires reversing the Na+/Ca2+ exchanger. During the pathological process, the chronic and acute excessive accumulation of ROS plays a significant role in the occurrence and development of HF (13). Excessive concentrations of ROS cause oxidative stress damage in cardiac cells, which subsequently disrupts the dynamic balance between free radical production and the antioxidant system. This imbalance in the antioxidant system can activate the mitochondrial-dependent apoptotic pathway, ultimately triggering programmed cell death in cardiac cells (14). Based on this, targeted regulation of the ROS signal cascade reaction, especially by restoring the balance of the antioxidant system to intervene in the pathological apoptotic process, has become a key therapeutic direction for improving the prognosis of cardiovascular diseases.

### 2.2 Myocardial fibrosis: an inevitable consequence of cardiomyocyte damage

When cardiomyocytes encounter damaging stimuli (e.g., ischemia, hypoxia, inflammation, and oxidative stress), a silent but irreversible remodeling process is initiated. Damaged cardiomyocytes release cytokines and growth signals, awakening dormant fibroblasts and promoting their transformation into myofibroblasts (15). These activated interstitial cells continuously secrete extracellular matrix (ECM) components such as type I and type III collagen, disrupting the dynamic balance of matrix synthesis and degradation in the original myocardial tissue (15, 16). As collagen fibers abnormally deposit in the myocardial interstitium and around blood vessels, a dense network of fibrous scars is formed. As a result, the myocardial tissue gradually loses its elasticity and compliance, and the stiffness of the ventricular wall significantly increases. This structural change initially causes diastolic dysfunction of the ventricle, manifested as restricted cardiac filling. Subsequently, due to the mechanical traction and electrical signal conduction interference exerted by fibrous scars on the surviving cardiomyocytes, the contractile function gradually deteriorates, resulting in a decrease in ejection fraction (16, 17). These dynamic remodeling processes of the ECM are crucial for the mechanical support, signal transduction, and intercellular interactions of myocardial tissue. Their imbalance directly aggravates the deterioration of ventricular geometry and function, profoundly affecting the clinical prognosis of patients. The following text will systematically elaborate on the occurrence and key regulatory mechanisms of myocardial fibrosis and focus on the emerging molecule secreted frizzled-related protein 1 (Sfrp1), which plays a significant role in inhibiting fibrosis and delaying the progression of HF.

#### 2.2.1 The core significance of chronic HF and ventricular remodeling

The occurrence and development of chronic HF (CHF) are not only caused by the progressive abnormalities of the structure and function of cardiac muscle cells but are also significantly influenced by the remarkable changes in the ECM (18, 19). The myocardial extracellular matrix is not merely a static supporting structure. Its dynamic changes in composition, content, and cross-linking status profoundly affect the mechanical properties and signal transduction of the heart. The pioneering research conducted by scholars such as Frangogiannis (20) and others has profoundly revealed the complex and precise regulatory mechanisms of the extracellular matrix of cardiac muscle cells. Their work has significantly expanded people's understanding of the causes of heart diseases, particularly HF, providing a solid theoretical foundation for subsequent research and initiating a new journey in this field of study.

#### 2.2.2 The formation of myocardial fibrosis is related to the role of fibroblasts

Structural and functional remodeling of myocardial tissue, especially myocardial fibrosis, is the core pathological process through which various cardiovascular diseases progress to HF. Myocardial fibroblast activation is a key driving factor. Studies have shown that activated fibroblasts trigger significant remodeling of the ECM, leading to profound structural and functional changes in the myocardium (21).

The core aspect of this reconstruction lies in the imbalance of collagen metabolism. Activated fibroblasts secrete large amounts of primary collagen chains. These primary collagen molecules first self-assemble to form collagen fibrils. Subsequently, under the catalysis of lysyl oxidase (LOX), specific lysine and hydroxylysine residues in the original collagen molecule undergo oxidative deamination, generating aldehyde groups, thereby initiating the covalent cross-linking reaction between the molecules. As a crucial posttranslational modification step, collagen cross-linking plays a dual role. On the one hand, it can enhance the extensibility of the myocardial tissue, which is crucial for maintaining the normal diastolic function of the heart. On the other hand, cross-linking significantly enhances the ability of collagen fibers to resist degradation by matrix metalloproteinases (MMPs), thereby helping to maintain the structural integrity of the cardiac matrix and counteract pathological ventricular dilation (22).

However, once this intrinsic protective mechanism becomes dysregulated, it yields opposing outcomes. Excessive collagen synthesis and deposition, particularly when accompanied by insufficient degradation, result in the formation of dense fibrotic scars. These scars not only fail to confer functional improvement but also exacerbate ventricular wall stiffness, perpetuating the vicious cycle of ventricular remodeling and ultimately driving a significant increase in HF risk (23). Therefore, the hypertrophy of cardiomyocytes, excessive pathological collagen deposition, and abnormal proliferation of the ECM—these three factors working together—constitute the typical pathological features of myocardial fibrosis (24).

In the pathological network of HF, oxidative stress-induced myocardial damage and excessive collagen deposition during fibrosis do not occur independently. Instead, they further trigger the three core mechanisms of “intracellular injury amplification loop” autophagy disorder of cardiomyocytes, abnormal apoptosis, and calcium metabolism disorder: Autophagy is the first line of defense to remove damaged organelles (such as damaged mitochondria due to oxidative stress). If autophagy fails, accumulated damage substances will activate apoptosis, and both of these will disrupt the regulation of calcium in mitochondria, leading to calcium overload and further exacerbating autophagy and apoptosis. The three mechanisms reinforce each other, accelerating the loss of cardiomyocytes and the impairment of contractile function and promoting the progression of myocardial injury to decompensated HF. The following paragraphs will respectively elaborate on the regulatory mechanisms of these three factors and their roles in HF.

### 2.3 Autophagic disorder in cardiomyocytes: mechanisms and their link to myocardial hypertrophy

In the field of cardiovascular disease research, numerous experiments have demonstrated that multiple key mechanisms are closely linked to the status of cardiomyocytes (25–27). Studies indicate that autophagy exerts a pivotal role in the pathogenesis of myocardial hypertrophy. In pressure overload-induced myocardial hypertrophy models, autophagy is suppressed, and its activation may potentially attenuate the progression of myocardial hypertrophy (28, 29).

Autophagy is a core mechanism for maintaining cellular homeostasis. Via the “autophagosome–lysosome” axis, it selectively degrades damaged organelles (e.g., mitochondria), misfolded proteins, and pathogens, maintaining a dynamic balance between material circulation and energy metabolism (30). This process begins with the activation of the autophagy initiation complex (ULK1-FIP200-Atg13), which subsequently forms a double-membrane structure known as an autophagosome. After enclosing the target components, it fuses with the lysosome and undergoes degradation through the action of acidic hydrolases (31).

Autophagy, as the “garbage disposal system” of cells, maintains myocardial homeostasis by eliminating abnormal proteins related to fibrosis and repairing damaged mitochondria due to oxidative stress. In HF, autophagy balance is disrupted: Oxidative stress exceeds autophagy’s clearance capacity, and RAAS activation induces AngII, which inhibits autophagic flux. This forms a “damage accumulation–autophagy inhibition” vicious cycle, directly promoting myocardial hypertrophy and functional impairment (32).

In cardiac muscle cells, mitophagy is particularly crucial for sustaining mitochondrial quality control and efficient energy metabolism. Canonically, it mediates the selective clearance of damaged mitochondria via well-characterized receptor-dependent pathways, including the PINK1–Parkin pathway, BNIP3/NIX pathway, and FUNDC1-dependent pathway. Specifically, FUNDC1—a key mitophagic receptor in cardiomyocytes—recognizes impaired mitochondria and facilitates the binding of LC3 protein between the outer mitochondrial membrane and the autophagosomal membrane, thereby enabling the targeted sequestration and degradation of damaged mitochondrial components. Beyond these classical mitophagic routes, recent studies, as highlighted in the study by Wang et al. (33), have demonstrated that damaged mitochondria can also be cleared through alternative mechanisms, including mitochondria-derived vesicles (MDVs), exopheres, and extracellular vesicles (EVs); these pathways act as complementary routes to further support mitochondrial quality maintenance in cardiac muscle cells (34, 35). Under pathological conditions, the AngII produced by the activation of the renin–angiotensin system (RAAS) becomes an important factor contributing to myocardial hypertrophy (36). It should be noted that when autophagy fails to remove damaged mitochondria, it not only disrupts energy metabolism but also releases apoptotic factors such as cytochrome c, directly activating the apoptotic pathway of cardiomyocytes. This “autophagy failure → mitochondrial damage → apoptosis activation” connection indicates that autophagy disorder is often the prerequisite for excessive cardiomyocyte apoptosis in HF and also links autophagy with the subsequent pathological process of cell death.

It is worth emphasizing that autophagy plays a crucial role in connecting the quality control of mitochondria with apoptosis. When the function of autophagy (especially mitochondrial autophagy) is impaired, the accumulation of damaged mitochondria not only leads to metabolic disorders but also directly activates the mitochondrial apoptosis pathway mediated by Bcl-2/Bax by releasing apoptotic factors such as cytochrome c, making autophagy disorder an important initiating factor for excessive apoptosis of cardiac muscle cells.

### 2.4 Abnormal apoptosis of cardiomyocytes

Excess apoptosis is triggered by autophagy failure (Section 1.3) and interacts with myocardial fibrosis. Apoptotic bodies from apoptotic cardiomyocytes are engulfed by fibroblasts, activating them to secrete extracellular matrix and promote collagen deposition (37). Excessive activation of apoptosis in HF disrupts the balance between cardiomyocyte loss and repair, directly reducing the number of functional cardiomyocytes and indirectly aggravating fibrosis. At the same time, its core mitochondrial apoptotic pathway also regulates calcium metabolism, laying the foundation for subsequent calcium homeostasis disorders.

Abnormal apoptosis of cardiomyocytes is also closely related to cardiovascular diseases. For instance, when a myocardial infarction occurs, ischemia and hypoxia can trigger apoptosis of cardiomyocytes, which in turn leads to a decline in cardiac function. Furthermore, mitochondria play a central role in cell apoptosis. When cells receive apoptotic signals, the permeability of the mitochondrial membrane changes, leading to the release of apoptotic factors such as cytochrome c, which initiates the apoptotic process.

#### 2.4.1 Myocardial apoptosis: the turning point from physiological balance to pathological remodeling

The apoptosis of cardiac muscle cells is crucial for maintaining homeostasis in the heart. It enables the thinning of the ventricular wall during adolescence and helps renew the myocardium after exercise, both of which rely on the precise regulation of cell numbers. However, when pathological factors such as coronary heart disease and hypertension come into play, apoptosis transforms from a “trimming tool” into a “destroyer.” Take acute myocardial infarction as an example. At the edge of the infarcted area, the surviving cardiomyocytes trigger the mitochondrial apoptosis pathway due to ischemia and hypoxia: Pro-apoptotic proteins Bax/Bak insert into the outer mitochondrial membrane, forming a permeability transition pore, releasing cytochrome c and apoptosis-inducing factor, activating the Caspase-9/-3 cascade reaction, leading to “orderly suicide” of cardiomyocytes. This type of apoptosis can be detected within 24 h after the infarction and persists for several weeks, eventually leading to an expansion of the infarcted area. What is even more worthy of vigilance is the myocardial remodeling triggered by apoptosis. The surviving myocardium compensates for the lost cells by activating the sympathetic nerve–renin–angiotensin system (RAAS), resulting in cell hypertrophy and interstitial fibrosis. The echocardiogram showed that 6 months after the infarction, the left ventricular end-diastolic volume could increase, accompanied by the remodeling of gap junctions—the originally orderly arranged intercalated discs were separated by fibrosis, and the conduction speed of electrical signals decreased, leading to ventricular arrhythmias. Clinical observations revealed that approximately 30% of patients with myocardial infarction had no HF during the acute phase but experienced a decrease in ejection fraction 6 months later due to the accumulation of apoptosis, which is a typical manifestation of the vicious cycle of apoptosis repair.

#### 2.4.2 Excessive apoptosis: the key driver of cardiac function decline

The irreversibility of cardiomyocytes makes apoptosis an irreversible “cellular subtraction process.” Oxidative stress (e.g., hypertension) and endoplasmic reticulum stress (e.g., diabetic cardiomyopathy) continuously activate the apoptotic pathway, leading to cardiomyocyte loss (38). This “hidden loss” impairs cardiac function via three mechanisms. (1) Structural damage: After apoptotic bodies are phagocytosed, fibroblasts fill the vacancies, increasing collagen deposition and myocardial stiffness, which first impairs diastolic function. (2) Energy metabolism disorder: Fragmentation of mitochondria in apoptotic cells leads to reduced ATP production and depletion of contraction reserve. (3) Electrophysiological heterogeneity: The action potential duration of cells around the apoptotic area is prolonged, forming a reentrant circuit. The incidence of ventricular tachycardia in patients with HF is several times higher than that in normal individuals. The widely used RAAS inhibitors in clinical practice (such as angiotensin receptor antagonists) not only lower blood pressure but also have a more significant effect of blocking the activation of Bax induced by AngII, thereby reducing the apoptosis rate. These findings suggest that inhibiting excessive apoptosis and maintaining the number of cardiac cells may be the key to delaying cardiac aging.

#### 2.4.3 The crucial role of mitochondria in cell apoptosis

Mitochondria serve as the “energy factories” of cells and play a crucial regulatory role in the apoptosis process of vertebrate cells. Apoptosis is a vital biological process that helps maintain the organism's homeostasis. The mitochondrial-dependent apoptosis pathway is a central component of this precise regulatory network.

##### 2.4.3.1 Activation and mechanism of apoptotic protein Bax

In the intricate network governing cell fate, members of the Bcl-2 protein family, as key regulators of mitochondrial apoptosis, precisely orchestrate cellular life–death decisions through a complex protein–protein interaction network. This family is divided into two opposing subgroups: pro-apoptotic proteins, such as Bax and Bak, and anti-apoptotic members, including Bcl-2 and Bcl-xL. These proteins are broadly distributed across biological membrane systems, including the outer mitochondrial membrane, endoplasmic reticulum, and nuclear envelope (39). Forming a multi-level and multidimensional regulatory network plays a crucial role in maintaining the dynamic balance between cell survival and death.

Among them, the Bax protein, as a key executor of the apoptotic pathway, has a very specific regulatory mechanism. In the resting state of the cell, Bax is anchored to the outer mitochondrial membrane in a monomeric form, with its N-terminal domain closed and in a non-activated state. When cells are stimulated by apoptotic signals (e.g., oxidative stress and DNA damage), BH3-only proteins (e.g., Bid and Bim) act as signal sensors. They specifically bind to Bax's BH3 domain, inducing conformational changes and exposing its N-terminal hydrophobic domain. This conformational change triggers the oligomerization process of Bax, and multiple Bax monomers assemble to form a transmembrane pore (40). These channels work in synergy with the mitochondrial membrane permeability transition pore (MPTP), causing the MPTP to open abnormally. This leads to a rapid disintegration of the mitochondrial membrane potential, subsequently triggering the release of apoptotic factors such as cytochrome c (Cyt c) from the mitochondrial intermembrane space into the cytoplasm (41). The released Cyt c binds to Apaf-1 and dATP in the cytoplasm, recruits and activates Caspase-9, and ultimately triggers the downstream caspase cascade reaction, leading the cell toward apoptosis.

Multiple studies have confirmed that the expression level of Bax is significantly positively correlated with the degree of cell apoptosis. In the research of neurodegenerative diseases, overexpressing Bax through gene editing technology can accelerate the disintegration of mitochondrial membrane potential in neurons, significantly increase the release of cytochrome c, thereby exacerbating neuronal apoptosis and accelerating the disease progression. However, in tumor cells, using RNA interference technology to knock down the expression of the Bax gene can effectively inhibit the change in mitochondrial membrane permeability and significantly reduce the mortality rate of cancer cells (42). These findings further reveal the central role of Bax in regulating cell life and death, offering a significant theoretical basis for the development of disease treatment strategies targeting Bax.

##### 2.4.3.2 The inhibitory mechanism of anti-apoptotic protein Bcl-2

The anti-apoptotic protein Bcl-2 plays a central role in regulating cell apoptosis. The key mechanism lies in antagonizing the function of the pro-apoptotic protein Bax. Specifically, Bcl-2 effectively inhibits the oligomerization process of Bax by directly binding to it. The activation and oligomerization of Bax are the key steps for its formation of pores on the outer mitochondrial membrane, which allows the leakage of contents from the mitochondrial intermembrane space. Therefore, Bcl-2 directly inhibits the transformation of mitochondrial membrane permeability [mitochondrial outer membrane permeabilization (MOMP)] by blocking the oligomerization of Bax (43).

It is precisely through these two mechanisms (inhibiting the oligomerization of Bax and stabilizing the VDAC/mitochondrial membrane structure) that Bcl-2 effectively prevents the massive release of Cyt c from the mitochondrial intermembrane space into the cytoplasm (44). Once Cyt c is released into the cytoplasm, it acts as a key junctional molecule and binds to Apaf-1, an apoptotic protease-activating factor. In the presence of dATP/ATP, it induces conformational changes and oligomerization of Apaf-1, forming a large complex known as the apoptosome (45). This platform for apoptotic bodies can recruit and activate the inactive precursor of cysteine aspartic protease-9, known as Procaspase-9. This activation process allows Procaspase-9 to undergo self-cleavage, transforming it into the active form, Caspase-9. Once activated, Caspase-9 cleaves and activates key executors of apoptosis, specifically Caspase-3 and Caspase-7. This cascade of events initiates an irreversible series of protein hydrolysis reactions, ultimately leading to the characteristic morphological and biochemical changes associated with cell apoptosis.

Therefore, the Bcl-2 protein fundamentally blocks the release of Cyt c, which is the core initiating event of the apoptotic signaling pathway, by maintaining the integrity of the outer mitochondrial membrane. This mechanism is equivalent to cutting off the cascade reaction that leads to irreversible apoptosis at its source. This precise regulation of the mitochondrial apoptotic pathway makes Bcl-2 a crucial molecular switch that determines the fate of the cell (survival or apoptosis).

Mitochondrial dysfunction during apoptosis (such as abnormal membrane potential and opening of the permeability transition pore) directly damages the mitochondrial calcium uniporter (MCU) and the sodium–calcium exchanger, thereby disrupting calcium homeostasis (46). After the mitochondria lose their calcium-buffering capacity, cytoplasmic calcium overload activates calcium-dependent kinases (such as CaMKII), interfering with the excitation–contraction coupling. This process of “apoptotic mitochondrial dysfunction → impaired calcium regulation” indicates that calcium metabolism disorder is a downstream consequence of apoptosis, and calcium overload, in turn, exacerbates mitochondrial damage, forming a closed loop between apoptosis and calcium metabolism.

During apoptosis, the collapse of mitochondrial membrane potential and the abnormal opening of MPTP channels are not only the core events of apoptosis progression but also directly damage the functions of MCU and sodium–calcium exchanger, resulting in the loss of mitochondrial calcium-buffering capacity. This “apoptosis-related mitochondrial dysfunction” will disrupt the calcium homeostasis of cells, laying the pathological foundation for subsequent abnormal calcium metabolism and myocardial function damage.

### 2.5 Abnormal calcium metabolism and abnormal myocardial function

As mentioned earlier, autophagy disorders affect the calcium reabsorption function of the sarcoplasmic reticulum (SR), while abnormal apoptosis disrupts the calcium-buffering system of mitochondria. Together, these two factors contribute to the calcium metabolism imbalance in the state of heart failure. Moreover, the abnormal calcium metabolism is not merely a passive downstream consequence; the calcium overload it triggers will also cleave autophagy-related proteins through calcium-dependent proteases (such as calpain) and promote the oligomerization of Bax, thereby reversing the amplification of autophagy disorders and excessive apoptosis, forming a pathological closed loop of “autophagy–apoptosis–calcium metabolism,” which accelerates the process of myocardial remodeling.

Abnormal calcium metabolism is not an independent pathological process in HF but the “final common pathway” of autophagic dysregulation, apoptosis, and oxidative stress. Autophagy failure impairs sarcoplasmic reticulum calcium reabsorption, apoptosis damages mitochondrial calcium buffering, and excessive ROS modifies calcium channels (e.g., RyR2) to increase calcium leakage. Calcium overload not only directly disrupts excitation–contraction coupling and impairs myocardial systolic and diastolic functions but also reactivates excessive autophagy and apoptosis (e.g., calcium-dependent calpain cleaves autophagy-related proteins and promotes Bax oligomerization), thus becoming a key node in amplifying myocardial damage and accelerating the progression of HF (47).

The research conducted by Wikins et al. (108) has confirmed the role of Ca^2+^ as a second messenger in the occurrence of myocardial hypertrophy (48). Calmodulin Ⅰ (CaMⅠ) is a key calcium-binding protein. Cumulative evidence indicates that CaMⅠ can form a Ca^2^-CaMⅠ complex with Ca^2+^, and this complex can induce myocardial hypertrophy via multiple signal transduction pathways (49).

#### 2.5.1 Regulatory characteristics of the calcium/calcineurin-dependent protein kinase family

The calcium/calmodulin-dependent protein kinase family (CaMKs), as key intracellular mediators of calcium signal transduction, exhibits unique regulatory characteristics during the pathological process of myocardial remodeling (50). Myocardial remodeling involves complex pathological changes such as hypertrophy of cardiomyocytes, apoptosis, and interstitial fibrosis. CaMKs can deeply participate in the regulation of this process by sensing fluctuations in intracellular calcium signals (51, 52). When cardiac muscle cells are subjected to mechanical stress or stimulated by neurohumoral factors, the intracellular calcium homeostasis is disrupted. CaMKs are rapidly activated, becoming a key node in the calcium signal transduction network, converting calcium signals into downstream molecular events, and influencing the structural and functional remodeling of cardiac muscle cells.

Among the members of this family, the CaMKII and CaMKIV subtypes both exhibit nuclear localization characteristics, enabling them to shuttle into the nucleus and directly regulate gene transcription activities (53). Both achieve signal decoding through their unique autophosphorylation properties: When the intracellular calcium ion concentration undergoes periodic fluctuations (such as the calcium transient during the excitation–contraction cycle of cardiac muscle cells), CaMKII and CaMKIV can bind to calmodulin (CaM), triggering the phosphorylation of their specific sites, resulting in a continuously activated state (i.e., “autophosphorylation memory”) (51). This modification enables the kinase to precisely identify the temporal parameters of calcium ion fluctuations (including amplitude, frequency, and duration)—for instance, high-frequency calcium fluctuations can drive CaMKII to phosphorylate downstream substrates more efficiently, whereas low-frequency fluctuations may activate different substrate preferences, thereby regulating the gene transcriptional activity of intracellular calcium response elements [such as cAMP response elements (CRE) and NFAT binding elements] and affecting the expression of genes related to myocardial remodeling (such as myocardial hypertrophy-related genes ANP and BNP, and fibrosis-related genes Col1A1) (51, 52).

It is essential to note that different subtypes have significant differences in their functions within the cardiovascular system. CaMKII, a key regulatory factor, plays a vital role in maintaining the electrical homeostasis of the heart. It is central to the excitation–contraction coupling process because it phosphorylates target proteins, including the ryanodine receptor 2 (RyR2, which functions as a calcium release channel in the sarcoplasmic reticulum) and L-type calcium channels, such as the Cav1.2 subunit (54). When CaMKII phosphorylates RyR2, it can increase the probability of channel opening, leading to calcium leakage from the sarcoplasmic reticulum and affecting myocardial contractility and relaxation functions; the phosphorylation of L-type calcium channels will change the channel kinetics, regulate the amplitude and duration of calcium influx, and thereby affect the morphology of action potentials and cardiac rhythm (54, 55). A large number of studies have confirmed that in diseases such as arrhythmias (such as ventricular arrhythmias) and HF, the excessive activation of CaMKII is an important mechanism that promotes myocardial electrical remodeling and contractile dysfunction (51, 52). The role of CaMKIV in myocardial hypertrophy is still a subject of debate (56). Some studies have found that CaMKIV can enhance the transcriptional activity of cAMP response element-binding protein (CREB) by phosphorylating it and can upregulate the expression of hypertrophic genes. It plays a role in promoting hypertrophy in models of myocardial hypertrophy induced by pressure load or neuroendocrine factors (57). However, accumulating studies have indicated that CaMKIV may exert an inhibitory effect on myocardial hypertrophy through interactions with other signaling pathways, most notably the mitogen-activated protein kinase (MAPK) pathway. Its specific functions are modulated by multiple factors, including the cellular microenvironment, types of pathological stimuli, heterogeneity among cardiomyocyte subtypes, and expression levels of upstream/downstream regulatory molecules. Further in-depth investigations are required to elucidate its precise role and underlying mechanisms in myocardial hypertrophy.

#### 2.5.2 Association between abnormal CREB phosphorylation and myocardial hypertrophy

The nuclear transcription factor CREB is an essential regulator of transcription within the cell. Abnormal phosphorylation of this factor plays a significant role in regulating calcium imbalances and the expression of myocardial hypertrophy (58). In the physiological activities of cardiac muscle cells, calcium signals precisely control the transcription of downstream target genes (such as genes related to cardiac hypertrophy and energy metabolism) by regulating the phosphorylation state of CREB. When calcium metabolism disorders occur (such as pathological calcium overload and abnormal calcium oscillation patterns), the balance of the CREB phosphorylation process is disrupted, which can drive the abnormal high expression of genes related to cardiomyocyte hypertrophy (such as ANP, BNP, and β-MHC), promoting the occurrence and development of myocardial hypertrophy (59).

### 2.6 Summary of the core pathological mechanism network of HF

The core pathological mechanism network of HF is a complex and interconnected cascade centered on cardiomyocyte damage, mainly composed of five interrelated core links, namely, oxidative stress, myocardial fibrosis, autophagy disorder, abnormal apoptosis, and calcium metabolism disorder, which together form a “damage–amplification–decompensation” vicious cycle. Oxidative stress is the initial trigger, and excessive ROS not only directly damages cardiomyocyte structure but also induces mitochondrial dysfunction, which in turn promotes the activation of fibroblasts and initiates myocardial fibrosis. Myocardial fibrosis increases ventricular stiffness and further impairs cardiac function, while also providing a pathological microenvironment for the occurrence of autophagy disorder and abnormal apoptosis. Autophagy disorder, as a key link in the “injury amplification loop,” fails to clear damaged organelles such as mitochondria, leading to the accumulation of cytotoxic substances and directly activating the mitochondrial apoptotic pathway mediated by the Bax/Bcl-2 balance. Abnormal apoptosis reduces the number of functional cardiomyocytes, and the apoptotic bodies further activate fibroblasts to aggravate fibrosis, forming a mutual promotion loop between apoptosis and fibrosis. Calcium metabolism disorder is the final common pathway of the above mechanisms: Autophagy disorder impairs sarcoplasmic reticulum calcium reabsorption, apoptosis damages mitochondrial calcium-buffering capacity, and oxidative stress modifies calcium channels, all of which lead to calcium overload. Calcium overload in turn activates calcium-dependent kinases to exacerbate autophagy disorder, apoptosis, and myocardial hypertrophy, thereby amplifying the entire pathological network and ultimately promoting the progression of myocardial injury to decompensated HF.

## 3 The structural characteristics and functions of Sfrp1, and the relationship between Sfrp1 and cardiomyocyte damage and HF

### 3.1 The structural characteristics and functions of Sfrp1

**Figure 1:**
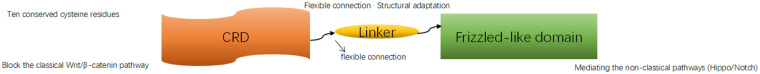
Sfrp1 Molecular Structure Diagram. Diagram showing the relationship between CRD, a linker, and a Frizzled-like domain. CRD contains ten conserved cysteine residues and blocks the Wnt/β-catenin pathway. The linker provides a ﬂexible connection for structural adaptation. The Frizzled-like domain mediates non- classical pathways such as Hippo and Notch.

Its modular structural design enables Sfrp1 to specifically bind to different ligands and participate in complex signal regulation. The CRD is the most distinctive structural domain of Sfrp1. It is highly homologous to the CRD in the Wnt protein and contains 10 conserved cysteine residues. It maintains a stable conformation by forming specific disulfide bonds, which are the core region for the binding of Sfrp1 to the Wnt protein (60). The Frizzled domain structure is similar to the transmembrane domain of the Frizzled receptor. Although Sfrp1 lacks the transmembrane part, this structure can interact with other receptors on the cell membrane and mediate signal transmission. The middle connecting region plays the role of a flexible connection, giving Sfrp1 a flexible structure and enabling it to better bind to different molecules (Figure 1) (61).

Sfrp1's structural characteristics make it a natural antagonist of the Wnt signaling pathway. Via its CRD, it binds to Wnt proteins, preventing their interaction with Frizzled receptors and LRP5/6 co-receptors, thus inhibiting classical Wnt/β-catenin pathway activation (22). Furthermore, Sfrp1 can also regulate the non-classical Wnt signaling pathway by binding to other membrane receptors and plays a key regulatory role in processes such as cell proliferation, differentiation, migration, and maintenance of tissue homeostasis. During the pathological process of HF, the structural characteristics of Sfrp1 enable it to participate in regulating processes, such as cardiomyocyte apoptosis and fibrosis, and have a significant impact on the progression of the disease.

Given the central role of ventricular remodeling and ECM dysregulation in the pathogenesis of heart failure, research has increasingly focused on endogenous molecules capable of modulating these core processes. Among these, Sfrp1 has emerged as a key regulatory factor that confers cardioprotection by simultaneously targeting multiple facets of this pathological network (62).

### 3.2 The relationship between Sfrp1 and cardiomyocyte damage and HF

Cardiomyocyte damage serves as the initiating factor of HF, and these two elements form a vicious cycle characterized by “damage remodeling decompensation.” Therefore, protecting cardiomyocytes represents a key strategy to delay the progression of HF. Cardiomyocytes are the fundamental functional units that constitute the cardiac structure; through effective contraction and relaxation, they facilitate blood circulation and maintain the essential functions of the heart. Once cardiomyocytes are damaged, the heart's ability to pump blood to various tissues and organs throughout the body becomes impaired, which may ultimately lead to HF.

Secreted frizzled-related protein 1 (Sfrp1) is a secreted glycoprotein belonging to the Frizzled (Fz) family. Due to the lack of a transmembrane domain, it is primarily localized in the extracellular matrix. Its molecular structure contains a CRD that shares high homology with the CRD of the Frizzled receptor, enabling it to competitively bind to the Frizzled/lipoprotein receptor-related protein (LRP) receptor complex with Wnt ligands, thereby inhibiting the activation of the Wnt/β-catenin signaling pathway. Sfrp1 plays a crucial role in cardiac development, cardiomyocyte differentiation, and the maintenance of tissue homeostasis. It can alleviate cardiomyocyte damage through multiple mechanisms, including inhibiting oxidative stress, regulating autophagy and apoptosis, and delaying myocardial fibrosis, making it a potential novel target for HF intervention (63).

This review summarizes the molecular mechanisms by which Sfrp1 alleviates cardiomyocyte damage and delays HF progression, providing a theoretical basis for targeted therapy. We integrated domestic and international experimental studies (*in vitro* cell models, animal models, clinical correlation analyses), focusing on six key pathways regulated by Sfrp1: (1) competitive inhibition of Wnt/β-catenin signal transduction; (2) reduction of ROS production; (3) inhibition of pathological cardiomyocyte proliferation; (4) delay of collagen deposition and myocardial fibrosis; (5) activation of autophagy and regulation of the mitochondrial apoptotic pathway (downregulation of Bax/Cyt c/Caspase-3 and upregulation of Bcl-2); and (6) inhibition of CREB phosphorylation to ameliorate calcium metabolism disorders. By coordinately regulating these mechanisms, Sfrp1 significantly mitigates myocardial oxidative stress, apoptosis, fibrosis, and calcium overload and blocks the “injury–repair–dysfunction” process. Its core function originates from the inhibition of the Wnt/β-catenin pathway and extends to multiple interconnected pathways such as Hippo and Notch, highlighting its potential as a novel therapeutic target for HF (4, 62).

#### 3.2.1 Sfrp1 blocks the transduction of the classical Wnt/β-catenin signaling pathway

##### 3.2.1.1 The role of the Wnt/β-catenin signaling pathway in cardiovascular diseases

The upstream of the classical Wnt signaling pathway mainly includes Wnt ligands, Frizzled (Fz) receptors, and low-density LRP co-receptors (64). When Wnt ligands bind to Fz/LRP, they inhibit the activity of glycogen synthase kinase-3β (GSK-3β) downstream, preventing β-catenin from being phosphorylated and degraded (65). As a result, β-catenin accumulates in the cytoplasm and enters the nucleus. Inside the cell nucleus, β-catenin binds to T-cell factor/lymphoid enhancer factor (Tcf/Lef) transcription factors, activating the expression of target genes such as c-Myc and Cyclin D1 (66) and participating in processes such as cell proliferation, differentiation, and apoptosis. The abnormal activation of this pathway is closely related to the occurrence and development of various diseases. In cardiovascular diseases, excessive activation can lead to myocardial fibrosis, inflammatory responses, and cell apoptosis, thereby exacerbating HF (67). Specifically, this evolutionarily conserved signaling pathway not only participates in the temporal regulation of cardiomyocyte differentiation but also plays a key regulatory role in maintaining the homeostasis of the myocardial tissue microenvironment. The classical Wnt signaling system has distinct bidirectional regulatory characteristics during the development of the cardiovascular system and in the maintenance of mature organ functions. Under normal physiological conditions, moderate activation of this signaling pathway can promote the proliferation and survival of cardiomyocytes (68); however, in pathological conditions, excessive activation will lead to myocardial fibrosis, inflammatory responses, and cell apoptosis, thereby exacerbating HF.

##### 3.2.1.2 The mechanism by which Sfrp1 inhibits the activity of the Wnt/β-catenin signaling pathway

**Figure 2:**
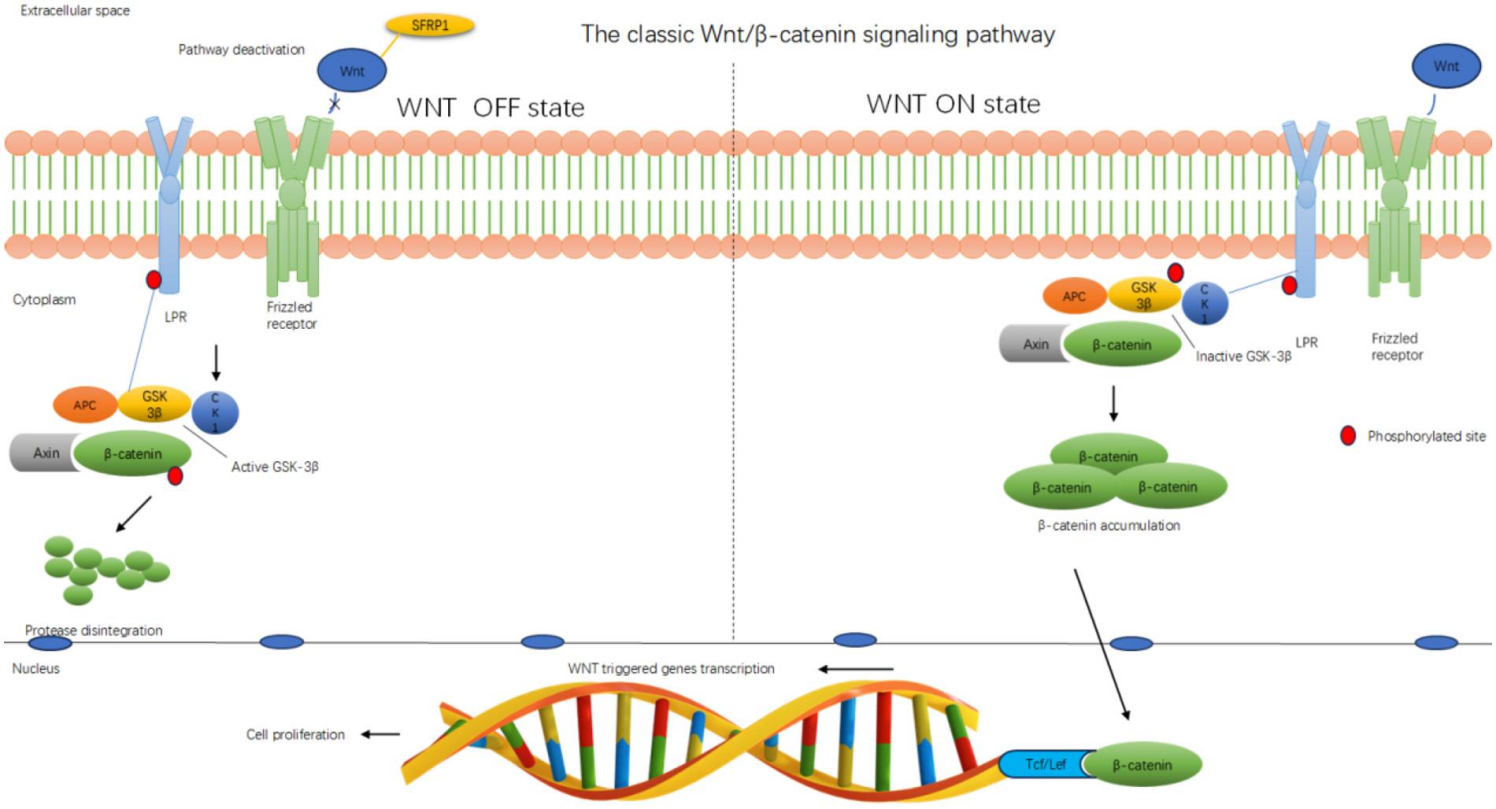
Sfrp1 inhibits the classical Wnt/β-catenin signaling pathway. Diagram illustrating the classic Wnt/β-catenin signaling pathway. The WNT OFF state shows pathway deactivation with SFRP1 inhibiting Wnt, leading to active GSK-3β degrading β-catenin. The WNT ON state shows Wnt binding to the receptor, inactivating GSK-3β and accumulating β-catenin, which translocates to the nucleus and interacts with Tcf/Lef for gene transcription, promoting cell proliferation. The membrane, cytoplasm, and nucleus are labeled. Red circles indicate phosphorylated sites.

During the progression of cardiovascular diseases, Sfrp1 acts as an inhibitor of the Wnt/β-catenin signaling pathway. It shares a similar CRD with the Frizzled receptor in the Wnt/β-catenin pathway. Therefore, Sfrp1 can compete with the coiled protein receptor for binding to the Wnt ligand on the plasma membrane, preventing β-catenin in the cytoplasm from entering the nucleus to activate the Tcf/Lef transcription factors, thereby shutting down the downstream biological effects and inhibiting the expression of the target gene, thus exerting a regulatory function (18, 69–71). Through analysis of the molecular structure characteristics, Sfrp1 is unable to anchor to the cell membrane surface due to the lack of transmembrane domains. Instead, it selectively combines with soluble Wnt ligands within the extracellular matrix and FZD receptors on the cell membrane surface (Figure 2). By forming a ternary competitive inhibitory complex, Sfrp1 can specifically antagonize the activation process of the classical Wnt/β-catenin signaling pathway, effectively blocking the downstream signal transduction cascade reaction (70). Pathophysiologically, this molecular antagonism significantly inhibits pathological cardiomyocyte hypertrophy and improves ultrastructural damage by reducing β-catenin nuclear translocation, ultimately delaying cardiac function decompensation (72).

#### 3.2.2 Sfrp1 inhibits the generation of oxygen free radicals

Research in redox biology has confirmed that ROS serve as important signaling molecules that help regulate cellular homeostasis within specific concentration thresholds. At physiological levels, ROS are essential for maintaining fundamental life processes, such as cell proliferation, differentiation, and metabolic adaptability, by effectively regulating intracellular signaling pathways involved in these activities, including signal transduction and apoptosis.

**Figure 3:**
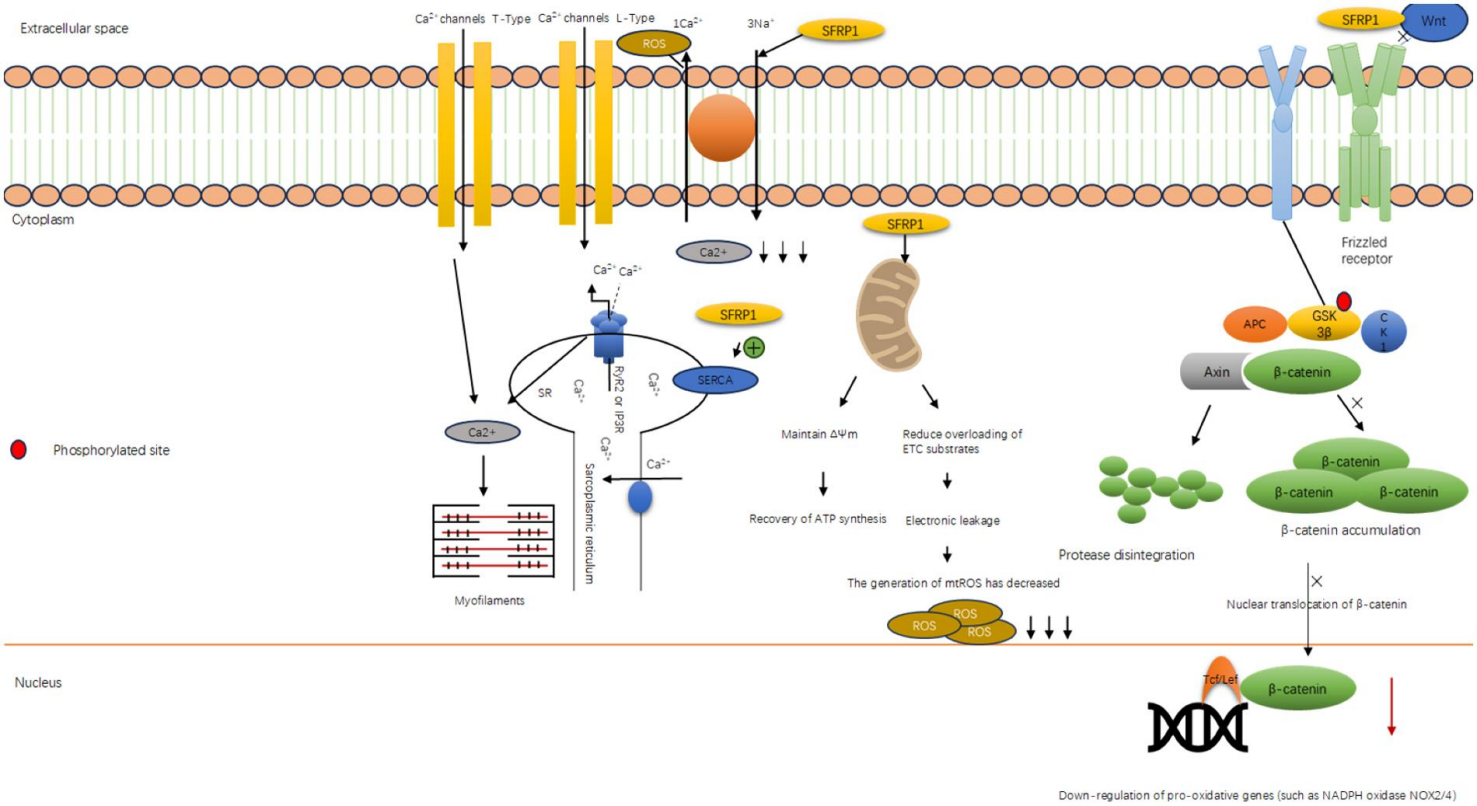
Sfrp1 inhibits the generation of oxygen free radicals. Diagram illustrating the classic Wnt/β-catenin signaling pathway. The WNT OFF state shows pathway deactivation with SFRP1 inhibiting Wnt, leading to active GSK-3β degrading β-catenin. The WNT ON state shows Wnt binding to the receptor, inactivating GSK-3β and accumulating β-catenin, which translocates to the nucleus and interacts with Tcf/Lef for gene transcription, promoting cell proliferation. The membrane, cytoplasm, and nucleus are labeled. Red circles indicate phosphorylated sites.

However, if ROS production exceeds the capacity of cells to eliminate it, it can lead to cellular and tissue damage, resulting in oxidative stress (73). The excessive production of reactive oxygen free radicals leads to an imbalance in the heart's antioxidant system, particularly in the context of the previously discussed pathological conditions. Targeted regulation of the ROS signaling pathway—most notably via restoration of antioxidant system balance—has emerged as a key therapeutic approach for intervening in the pathological process of apoptosis. This strategy aims to improve the prognosis for individuals with cardiovascular diseases (74). Some scholars have conducted research indicating that Sfrp1 is a key molecule in regulating the oxidative stress response (75). Under pathological conditions such as myocardial ischemia/reperfusion injury (I/R), the Wnt/β-catenin signaling pathway is often abnormally activated. The excessive activation of this pathway will promote mitochondrial dysfunction and upregulate the expression and activity of key pro-oxidative enzymes such as NADPH oxidase (NOX), leading to the explosive production of ROS within the cells. Sfrp1 effectively inhibits the activation of the downstream β-catenin signaling pathway by directly binding to the Wnt protein and blocking its binding to the cell membrane receptor Frizzled and the LRP5/6 complex (63, 76). Some researchers have conducted studies showing that in the myocardial ischemia/reperfusion (I/R) model, the overexpression of Sfrp1 significantly reduces the nuclear translocation of β-catenin and the expression of its downstream target genes, such as c-Myc and Cyclin D1. Additionally, Sfrp1 leads to a noticeable decrease in the levels of NOX2 and NOX4 proteins in myocardial tissue, resulting in lower mitochondrial superoxide production (see Figure 3). This evidence demonstrates that Sfrp1 inhibits the primary cellular source of ROS by suppressing the classical Wnt signaling pathway (63, 77).

#### 3.2.3 Sfrp1 selectively inhibits pathological proliferation of cardiac cells: targeting cardiomyocytes and fibroblasts via multi-pathway cross talk

Pathological cardiac cell proliferation, a key driver of ventricular remodeling in HF, mainly includes two harmful subtypes: (1) pathological cardiomyocyte proliferation (abnormal re-entry of mature cardiomyocytes into the cell cycle, leading to myocardial hypertrophy), and (2) activated cardiac fibroblast proliferation (differentiation into myofibroblasts, promoting collagen deposition). Secreted frizzled-related protein 1 (Sfrp1) exerts selective inhibitory effects on both subtypes by orchestrating the cross talk of Wnt/β-catenin, Hippo, and Notch signaling pathways, without interfering with physiological myocardial repair. The following sections elaborate on its regulatory mechanisms and experimental evidence.

Recent studies show that Sfrp1 binds to Wnt ligands, preventing Wnt/β-catenin pathway activation, and specifically inhibits pathological cardiomyocyte proliferation without affecting physiological myocardial regeneration (78, 79). This regulatory mechanism has opened up innovative therapeutic strategies for mitigating pathological myocardial remodeling while preserving the potential for myocardial tissue repair (78). Specifically, researchers found that overexpression of Sfrp1 in the hearts of adult mice reduced the rate of cardiomyocyte re-entry into the cell cycle—an effect directly associated with increased levels of phosphorylated β-catenin (Ser33/37, a modification that targets β-catenin for proteasomal degradation) (80). Using single-cell sequencing, this study further confirmed the dynamic expression of Sfrp1 during the critical window of cardiac development and highlighted its role in spatiotemporally regulating the cardiomyocyte cell cycle to constrain pathological proliferation. In a complementary study, Han et al. (109) established a neonatal rat model to investigate cardiac interstitial fibroblasts and found that overexpression of β-catenin (the key downstream effector of the Wnt pathway inhibited by Sfrp1) significantly enhanced the mitotic activity of pathological cardiac cells and promoted the transformation of fibroblasts into myofibroblasts—accompanied by increased expression of α-smooth muscle actin (α-SMA), a classic myofibroblast marker (81). This finding underscores that β-catenin is a central mediator of both pathological cardiomyocyte proliferation and fibrotic remodeling, further supporting Sfrp1's role as a negative regulator of this pro-pathological pathway. Notably, Sfrp1 expression increases significantly within 7 days after birth in rodents, which correlates with the natural decline in physiological cardiomyocyte proliferation capacity (a normal developmental process); this distinction clarifies that Sfrp1 inhibits pathological proliferation rather than impairing physiological myocardial regeneration, positioning it as a potential target for balancing cardiomyocyte proliferation and regeneration in disease (63, 79). In a preclinical study of chronic HF, researchers delivered the Sfrp1 gene via an AAV-9 vector into the tail vein of rats with HF. After 4 weeks of treatment, the Sfrp1-overexpressing group showed significant improvements in cardiac function, including increased left ventricular ejection fraction (LVEF) and short-axis shortening rate (LVFS), compared with the control group. Immunohistochemical analysis revealed that Sfrp1 overexpression significantly downregulated proliferating cell nuclear antigen (PCNA)—a marker of pathological cell proliferation—consistent with reduced pathological cardiomyocyte proliferation. Additionally, Sfrp1 decreased cardiomyocyte apoptosis and improved ventricular geometric remodeling [as evidenced by reduced left ventricular end-diastolic diameter (LVEDD)] by modulating the Wnt/β-catenin signaling pathway (72). Their research indicates that the level of Sfrp1 is significantly negatively correlated with LVEF, suggesting its important role in the process of myocardial remodeling. Moreover, the significantly elevated expression level of Sfrp1 in patients with HF demonstrates its crucial role under pathological conditions. Furthermore, the researchers also explored the interaction of Sfrp1 with other signaling pathways, such as the Notch and Hippo pathways. These studies have provided us with a new perspective for comprehensively understanding how Sfrp1 enables myocardial regeneration.

##### 3.2.3.1 Inhibiting pathological cardiomyocyte proliferation: Sfrp1 coordinates Wnt/β-catenin and Notch pathways

Mature cardiomyocytes have limited proliferative capacity under physiological conditions; however, pressure overload or neuroendocrine stimulation (e.g., AngII) hyperactivates the Wnt/β-catenin pathway, which not only promotes the expression of proliferation-related genes (c-Myc, Cyclin D1) but also suppresses the Notch pathway (by reducing the release of Notch ligand DLL1), thereby driving pathological cell cycle re-entry (80, 82).

Sfrp1 interrupts this process through dual regulation: (1) As a competitive antagonist of Wnt ligands, Sfrp1 binds to Wnt proteins via its CRD, preventing Wnt from interacting with Frizzled/LRP receptors. This inhibits β-catenin nuclear translocation, downregulating the transcription of c-Myc and Cyclin D1 and blocking the G1/S phase transition of cardiomyocytes (72). (2) By inhibiting Wnt/β-catenin, Sfrp1 relieves the suppressive effect of Wnt on the Notch pathway, promoting DLL1 secretion from adjacent cells. DLL1 binds to Notch1 receptors on cardiomyocytes, triggering γ-secretase-mediated cleavage of the Notch intracellular domain (NICD). NICD translocates to the nucleus and forms a complex with CSL [C promoter binding factor 1 (CBF1)/Suppressor of hairless (Su(H))/Lag-1)] transcription factors, upregulating the expression of Hes/Hey genes. These genes further downregulate proliferation-promoting factors (Akt, Cyclin D), inducing cardiomyocytes to exit the cell cycle and return to a quiescent state (83).

Experimental verification: In adult mouse models of pressure overload, overexpression of Sfrp1 significantly reduced the proportion of cardiomyocytes positive for PCNA (a cell cycle marker) and decreased LVEDD. Meanwhile, the nuclear translocation of NICD and the expression of Hes1 were increased, confirming the restoration of Notch-mediated cell cycle arrest (72, 84).

In the Hippo signaling pathway—another key regulator of cell proliferation—Sfrp1 enhances cell polarity and adhesion-related signals to initiate phosphorylation of MST1/2 (Mammalian Sterile 20-like Kinase 1/2), a critical upstream kinase in the pathway. Upon activation, MST1/2 further phosphorylates and activates LATS1/2 (Large Tumor Suppressor 1/2), which in turn phosphorylates the core Hippo effectors YAP (Yes-associated protein) and TAZ [WW domain-containing transcription regulator 1 (WWTR1)]. Phosphorylation of YAP/TAZ promotes their retention in the cytoplasm (preventing nuclear translocation) or degradation, leading to a significant reduction in nuclear YAP/TAZ levels (83). Since nuclear YAP/TAZ forms a complex with TEAD (TEA domain transcription factor) to drive the expression of proliferation-promoting genes [e.g., connective tissue growth factor (CTGF) and cysteine-rich protein 61 (CYR61)], the reduction in nuclear YAP/TAZ inhibits TEAD complex activity and downregulates these pro-proliferative genes. This ultimately suppresses proliferation signals in cardiomyocytes, specifically targeting pathological proliferation (e.g., in response to pressure overload or inflammation) without impairing physiological repair processes (83).

##### 3.2.3.2 Suppressing cardiac fibroblast proliferation and myofibroblast differentiation: Sfrp1 targets Wnt/β-catenin-Hippo cross talk

Sfrp1 also modulates the Notch pathway—known to regulate cell cycle arrest in mature cardiomyocytes—by first inhibiting the Wnt/β-catenin pathway. Physiologically, the Wnt/β-catenin pathway exerts an antagonistic effect on Notch signaling (i.e., active Wnt/β-catenin suppresses Notch activity); by competitively binding to Wnt ligands, Sfrp1 alleviates this Wnt-mediated inhibition of the Notch pathway (82). This relief promotes the release of Notch ligands [e.g., Delta-like ligand 1 (DLL1)], which bind to Notch receptors (e.g., Notch1) on cardiomyocytes. Ligand-receptor binding triggers cleavage of the Notch receptor by γ-secretase, releasing the Notch intracellular domain (NICD). NICD translocates to the nucleus and forms a complex with CSL transcription factors [CBF1 (C-promoter Binding Factor 1)/Su(H) (Suppressor of Hairless)/Lag-1], activating the expression of Hes (Hairy and Enhancer of Split homolog) and Hey (Hes-related family member) genes (82). The Hes/Hey genes then downregulate the expression of proliferation-promoting factors such as Akt (protein kinase B) and Cyclin D, inducing cardiomyocytes to exit the cell cycle and enter a quiescent state—specifically limiting pathological re-entry into the cell cycle (e.g., in HF) while preserving the potential for Notch-mediated physiological repair (82). (Note: “Hes/Hay” in the original text is corrected to “Hes/Hey” to align with standard nomenclature.)

Cardiac fibroblasts are activated by damaged cardiomyocyte-derived cytokines (e.g., TGF-β), and their proliferation and differentiation into myofibroblasts (marked by α-SMA) are critical for excessive collagen deposition (15, 21). The hyperactivation of the Wnt/β-catenin pathway is a core driver of this process: β-catenin nuclear translocation upregulates the transcription of fibroblast activation-related genes (α-SMA, Col1A1) and synergizes with YAP/TAZ (effectors of the Hippo pathway) to enhance pro-fibrotic signals (85).

Sfrp1 inhibits fibroblast proliferation through two synergistic mechanisms: (1) By blocking the Wnt/β-catenin pathway, Sfrp1 reduces the nuclear accumulation of β-catenin, directly downregulating the expression of α-SMA and procollagen genes (81). (2) Sfrp1 enhances cell polarity and N-cadherin-mediated cell adhesion, activating the upstream kinases MST1/2 of the Hippo pathway. Activated MST1/2 phosphorylates LATS1/2, which in turn phosphorylates YAP/TAZ, promoting their cytoplasmic retention and degradation. This reduces the nuclear YAP/TAZ-TEAD complex, further suppressing the transcription of proliferation-related genes (CTGF, CYR61) (82).

Experimental verification: In neonatal rat cardiac fibroblast models, overexpression of β-catenin (mimicking Wnt hyperactivation) significantly increased the mitotic index and α-SMA expression; however, co-overexpression of Sfrp1 reversed these changes, accompanied by increased phosphorylation of YAP (Ser127, a marker of cytoplasmic retention) (81). In a rat HF model treated with AAV9-Sfrp1, immunohistochemical staining showed reduced α-SMA-positive fibroblasts and collagen deposition in myocardial interstitium, confirming the inhibitory effect on fibroblast activation (86).

##### 3.2.3.3 The interaction among the three signaling pathways: Wnt/β-catenin, Notch, and Hippo

The Wnt/β-catenin, Notch, and Hippo pathways form a coordinated network that Sfrp1 modulates to inhibit pathological cardiomyocyte proliferation, with clear functional interactions between each pathway:

1. Wnt/β-catenin and Notch cross talk: In the absence of Sfrp1, the Wnt/β-catenin pathway is hyperactivated and directly inhibits the Notch pathway—specifically reducing the release of Notch ligands (e.g., DLL1) and blocking Notch receptor cleavage (87). This inhibition of Notch signaling removes the constraint on proliferation-promoting genes (e.g., Cyclin D), allowing pathological cardiomyocyte proliferation (82). By inhibiting Wnt/β-catenin signaling, Sfrp1 relieves this inhibitory pressure on the Notch pathway, enabling normal release of DLL1 and activation of Notch downstream signals (e.g., Hes/Hey expression). This restores Notch-mediated cell cycle arrest, counteracting pathological proliferation (82).

2. Wnt/β-catenin and Hippo synergy: The Hippo pathway's YAP/TAZ and the Wnt pathway's β-catenin share overlapping targets in the nucleus (e.g., c-Myc and Cyclin D1) and exert a synergistic effect to drive pro-proliferative gene expression. Wnt/β-catenin and Hippo synergy: The Hippo pathway's YAP/TAZ and the Wnt pathway's β-catenin share overlapping targets in the nucleus (e.g., c-Myc and Cyclin D1) and exert a synergistic effect to drive pro-proliferative gene expression (85). Sfrp1 targets both pathways to block this synergy: (1) In the Hippo pathway, Sfrp1 promotes YAP/TAZ phosphorylation and cytoplasmic retention, reducing nuclear YAP/TAZ levels (83). (2) In the Wnt pathway, Sfrp1 inhibits β-catenin nuclear translocation, preventing its binding to Tcf/Lef transcription factors (85). This dual inhibitory effect of Sfrp1 ultimately leads to a significant downregulation of gene expressions such as c-Myc, thereby enhancing the inhibitory effect on proliferation. The Wnt/β-catenin, Notch, and Hippo signaling pathways form a synergistic network through a dual mechanism of “cell cycle exit and proliferation signal blockade,” jointly inhibiting the pathological proliferation of cardiomyocytes. This dual inhibition significantly downregulates c-Myc, Cyclin D1, and other pro-proliferative genes, amplifying the suppression of pathological proliferation.

**Figure 4:**
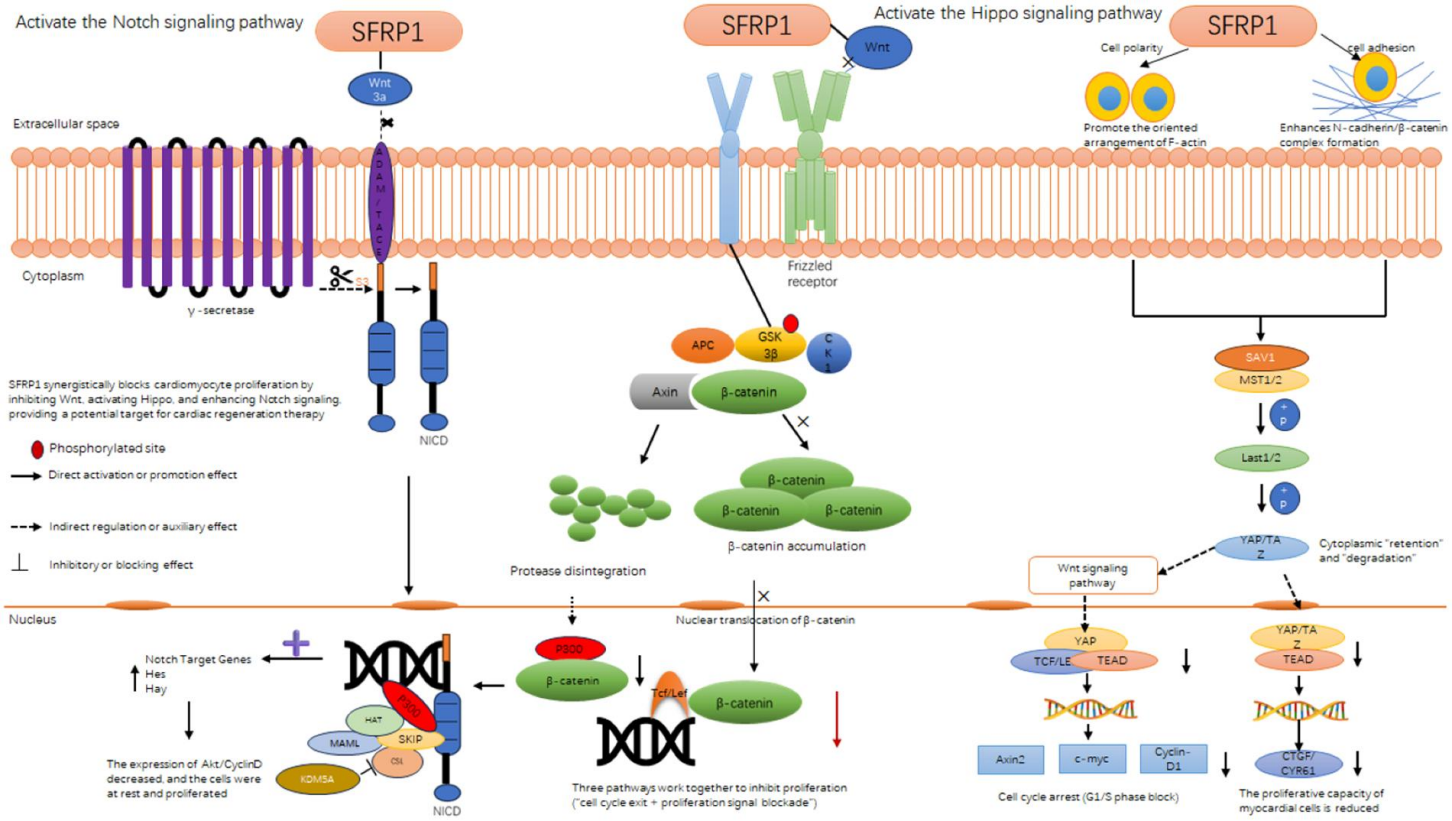
Sfrp1 inhibits cardiomyocyte proliferation through interactions with three signaling pathway. Diagram illustrating how SFRP1 interacts with and regulates signaling pathways including Notch, Wnt, and Hippo in cellular processes. It shows SFRP1's potential in blocking proliferation, highlighting actions like phosphorylation and β-catenin accumulation. The pathways influence cell adhesion, polarity, and gene transcription, impacting cardiac regeneration and myocardial cell proliferation.

Collectively, Sfrp1 coordinates the Wnt/β-catenin, Notch, and Hippo pathways through a “two-pronged mechanism”: (1) activating Notch to induce cell cycle exit and (2) blocking Wnt/Hippo-mediated proliferation signals. This synergistic network specifically targets pathological cardiomyocyte proliferation while avoiding interference with physiological myocardial repair, underscoring Sfrp1's potential as a therapeutic target for HF (Figure 4; Table 1).

**Table 1:** The interaction among the Wnt, Notch, and Hippo signaling pathways.

Interaction between pathways	Specific mechanism of action	Targeted cell type	The regulatory role of Sfrp1
Wnt ↔Notch	Wnt inhibits Notch, and SFRP1 indirectly activates Notch by inhibiting Wnt	Pathological cardiomyocytes	Relieve antagonism and activate Notch
Hippo ↔Wnt	YAP shares proliferative gene targets with β-catenin, and SFRP1 dual inhibits the nuclear functions of both	Cardiac fibroblasts	Synergistic enhancement of inhibitory effect
Multichannel integration	Hippo (YAP/TAZ), Notch (NICD), and Wnt (β-catenin) jointly regulate proliferation genes	Cardiomyocytes + fibroblasts	Form an anti-proliferation network

While significant progress has been made, current research on the regulatory mechanism of Sfrp1 in myocardial regeneration remains underdeveloped. Future research directions may include developing more efficient targeted delivery systems, exploring the interaction between Sfrp1 and other signaling pathways, and evaluating its safety and efficacy in clinical treatment. These studies will lay the foundation for advancing cardiac regeneration from basic research to clinical application.

Beyond inhibiting pathological proliferation, Sfrp1 also targets myocardial fibrosis—a key component of ventricular remodeling—by antagonizing the Wnt/β-catenin pathway, as elaborated in the following section.

#### 3.2.4 Sfrp1 delays myocardial fibrosis

CHF is a complex and progressive clinical syndrome. Its underlying pathophysiological processes extend beyond merely damaging cardiac muscle cells. Ventricular remodeling is recognized as a central factor in the onset and progression of CHF. The characteristic changes associated with ventricular remodeling include not only hypertrophy, apoptosis, and abnormal contraction of cardiac muscle cells but also significant alterations in the composition, structure, and function of the ECM surrounding these cells (18, 69). These dynamic remodeling processes of the ECM are crucial for the mechanical support, signal transduction, and intercellular interactions of the myocardial tissue. Their imbalance directly aggravates the deterioration of ventricular geometry and function, profoundly affecting the clinical prognosis of patients. The following text will systematically elaborate on the occurrence and key regulatory mechanisms of myocardial fibrosis and focus on the emerging molecule secreted frizzled-related protein 1 (Sfrp1), which plays a significant role in inhibiting fibrosis and delaying the progression of HF.

It is worth noting that during the remodeling process of the ECM, Sfrp1 was identified as an important regulatory factor. Sfrp1 participates in the regulatory network of myocardial fibrosis by antagonizing the Wnt signaling pathway (which plays a central role in development, tissue homeostasis, and fibrosis). Studies have shown that in the pathological conditions of excessive stress load, myocardial hypertrophy, and HF, the expression level of Sfrp1 often undergoes significant changes. The upregulation of Sfrp1 expression can inhibit the classical Wnt/β-catenin signaling pathway, and the excessive inhibition of this pathway has been proven to be closely related to the abnormal activation of fibroblasts and the pathological deposition of collagen (especially type I and type III collagen). Therefore, the dysregulation of Sfrp1 is one of the key molecular mechanisms that mediate the transformation of myocardial extracellular Matrix from adaptive remodeling to pathological fibrosis (88).

**Figure 5:**
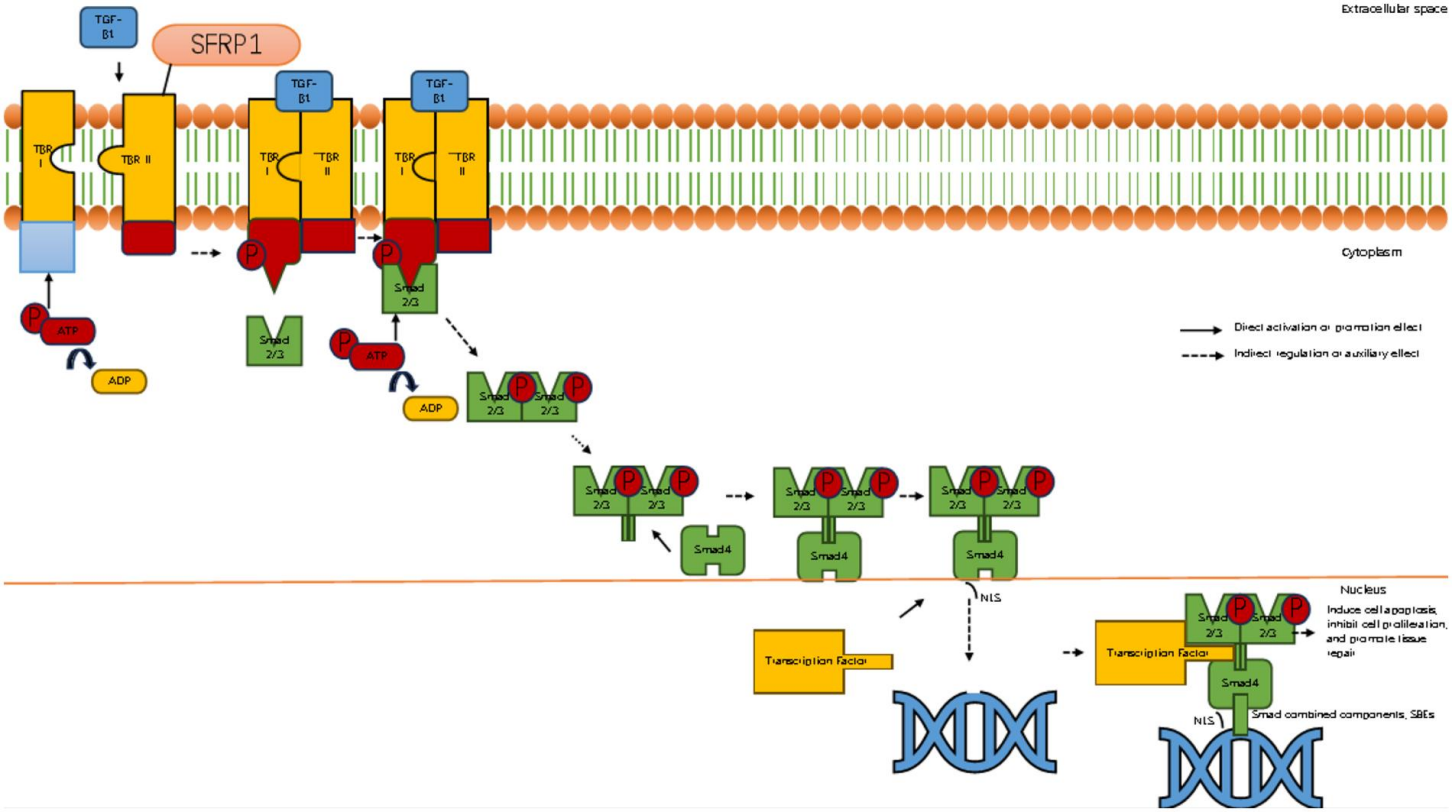
Sfrp1 inhibits myocardial fibrosis by suppressing the binding of TGF-β1 to its receptor. Diagram illustrating the TGF-β signaling pathway. It shows the binding of TGF-β1 to TBR I and II receptors on the cell membrane, phosphorylation of Smad proteins, and their translocation into the nucleus. The activated complex influences transcription factors affecting apoptosis, cell proliferation, and tissue repair. Components like ATP, ADP, and SFRP1 are also depicted. Arrows indicate direct and indirect regulation effects.

Given the significant role of myocardial fibrosis in the development of HF, intervention in the fibrosis process has become an important therapeutic target (89). In recent years, secreted frizzled-related protein 1 (Sfrp1) has attracted attention due to its crucial role in regulating the fibrosis signaling pathway. The research has found that Sfrp1, as an important secreted protein, can antagonize specific Wnt signaling pathways (such as Wnt/β-catenin). This pathway plays a central role in fibrotic processes, including activation, proliferation, and collagen synthesis of fibroblasts. Experimental evidence indicates that the expression level of Sfrp1 is often downregulated in cardiac fibrosis models. Supplementing externally or upregulating Sfrp1 internally can effectively inhibit the activation, proliferation, and collagen secretion of fibroblasts. Diminish the extent of myocardial fibrosis and enhance cardiac function. Therefore, in-depth exploration of the specific molecular mechanism by which Sfrp1 regulates myocardial fibrosis holds significant potential value for the development of new anti-fibrotic therapeutic strategies (Figure 5; Table 2).

**Table 2:** Summary of the key sites and mechanisms of Sfrp1 in inhibiting myocardial fibrosis.

Stage	Key mechanisms	Impact on myocardium	Sfrp1 intervention
Fibroblast activation	Triggered by injury/stress; initiates ECM remodeling	Begins structural remodeling	Inhibits fibroblast activation via Wnt signaling
Collagen synthesis	Activated fibroblasts secrete procollagen → assembles into fibrils	Increases collagen content in the ECM	Reduces procollagen gene expression
LOX-mediated cross-linking	LOX catalyzes covalent bonds between collagen molecules	Enhances tensile strength; balances ECM turnover	Suppresses LOX activity or expression
Pathological cross-linking	Excessive cross-links due to uncontrolled collagen deposition	Increases ventricular stiffness; impairs diastolic function	Disrupts pro-fibrotic signaling (e.g., Wnt/β-catenin)

#### 3.2.5 Initiate the autophagy process in cells, eliminate apoptotic cardiomyocytes, and regulate the mitochondrial apoptotic pathway

##### 3.2.5.1 Sfrp1 regulates autophagy to reduce myocardial hypertrophy

Some scholars have conducted research indicating that AngII stimulation can inhibit autophagy in cardiac muscle cells. Overexpression of the Sfrp1 gene promotes autophagy by blocking the Wnt/β-catenin signaling pathway, thereby alleviating myocardial hypertrophy (90). In mouse models subjected to pressure load or AngII stimulation, the ratio of autophagy marker protein LC3II/I decreases, and the p62 protein accumulates, suggesting that the autophagy flow is blocked (88). Further mechanism analysis reveals that AngII increases the expression of the catalytic subunit β5i of the immune proteasome. This subunit binds to the autophagy core protein ATG5, facilitating its ubiquitination and degradation, which impairs autophagy initiation. This leads to an exacerbation of myocardial fibrosis and cell hypertrophy due to the accumulation of damaged mitochondria and abnormal proteins (2). This compensatory imbalance of “ubiquitin-proteasome system (UPS) inhibition of autophagy” has also been confirmed in the myocardial tissues of patients with HF. The elevated levels of β5i are inversely related to the left ventricular ejection fraction (2). Regarding this pathological process, we have discovered that overexpression of the Sfrp1 gene can re-establish autophagy homeostasis by blocking the Wnt/β-catenin pathway. Sfrp1, as a secreted antagonist of Wnt ligands, competitively binds to Wnt ligands (rather than directly binding to LRP5/6), thereby preventing Wnt from interacting with Frizzled receptors and LRP5/6 co-receptors. This inhibits the nuclear translocation of β-catenin and relieves its transcriptional repression of autophagy-related genes (e.g., Atg5 and Beclin1) (88). In AngII-induced primary cardiomyocytes, Sfrp1 overexpression increased dephosphorylation of the mitochondrial autophagy receptor FUNDC1, promoted LC3-mitochondria colocalization, accelerated damaged mitochondria clearance, and reduced cell surface area by 32%. The expressions of hypertrophy markers such as ANP and BNP were significantly reduced (88, 91, 92). This protective effect was further enhanced in β5i knock-out mice, suggesting that the Sfrp1-Wnt pathway and the UPS-autophagy axis have an interactive regulatory relationship (2). It is worth noting that excessive activation of autophagy may also cause myocardial damage. Recent studies have found that circ-0001283, by stabilizing the MYL3 protein, abnormally activates the ERK-mediated autophagy signal, thereby exacerbating the hypertrophic phenotype (78). This indicates that there is a “threshold effect” for myocardial autophagy—that is, autophagy at physiological levels (such as the basal autophagy induced by 16 h fasting) exerts a protective effect (81), and pathological imbalance (either excessive inhibition or activation) can disrupt cellular homeostasis. Clinical data show that the autophagic flux state in hypertrophic myocardium is closely related to the classification of cardiac function. Patients with elevated BNP levels generally have abnormal autophagy markers (18). In conclusion, autophagy regulates myocardial remodeling through the dual functions of “removal and repair”: The AngII/β5i axis inhibits autophagy, leading to accumulation of damage, while the Sfrp1-Wnt pathway reconfigures the autophagic flow and alleviates hypertrophy. The imbalance of this dynamic equilibrium is a key node in the transformation from myocardial hypertrophy to HF. Targeting the β5i-ATG5 axis or the Sfrp1-Wnt pathway is expected to become a new strategy for intervening in myocardial remodeling (81, 88).

##### 3.2.5.2 The significance of Sfrp1 as an emerging regulatory factor and the experimental verification and mechanism of its inhibition of cardiomyocyte apoptosis

Recent studies have found that Sfrp1, as an important negative regulatory factor of the Wnt/β-catenin signaling pathway, has significant interactions with the mitochondrial apoptotic pathway (93, 94). Sfrp1 inhibits the nuclear movement of β-catenin by competitively binding to Wnt ligands, thereby regulating the expression of genes related to apoptosis. The latest research indicates that Sfrp1 can upregulate the expression of Bax protein and promote its conformational activation, while inhibiting the expression of Bcl-2. Ultimately, it accelerates cell apoptosis by enhancing mitochondrial membrane permeability (94). This regulatory mechanism plays a key role in pathological processes such as tumor occurrence and development, and neurodegenerative diseases, suggesting that Sfrp1 may serve as an important molecular bridge connecting Wnt signaling and mitochondrial apoptosis. The above studies have shown that the mitochondrial-dependent apoptotic pathway, through the precise regulation of Bcl-2 family proteins, constitutes the core mechanism for determining cell fate. Sfrp1, as an emerging regulatory factor, interacts with the mitochondrial apoptosis pathway, providing a new perspective for understanding the regulation of cell life and death. An in-depth study of this content may provide an important theoretical basis for the development of disease treatment strategies targeting the apoptosis pathway.

To explore whether the potential mechanism by which Sfrp1 inhibits cardiomyocyte apoptosis through regulating the Wnt/β-catenin signaling pathway involves a mitochondrial-dependent apoptotic pathway, some researchers established a systematic experimental verification system. Through quantitative analysis of apoptosis-related proteins in rat myocardial tissues, they focused on detecting the dynamic changes of Bcl-2 family members (Bax, Bcl-2) and apoptotic execution factors (activated Caspase-3). The experimental data showed that in the Sfrp1 overexpression model, the inhibitory effect of the Wnt/β-catenin signaling pathway was significantly correlated with the apoptotic regulatory proteins: The expression of Bax protein was significantly downregulated, while the level of Bcl-2 protein showed compensatory upregulation, resulting in a decrease in the ratio of pro-apoptotic to anti-apoptotic (Bax/Bcl-2). At the same time, the expression level of the apoptotic terminal effector molecule, activated Caspase-3, decreased synchronously. However, after restoring the activity of the Wnt/β-catenin pathway, the above protein expression profiles reversed, with the Bax/Bcl-2 ratio and the activation level of Caspase-3 returning to the basal state (90).

**Figure 6:**
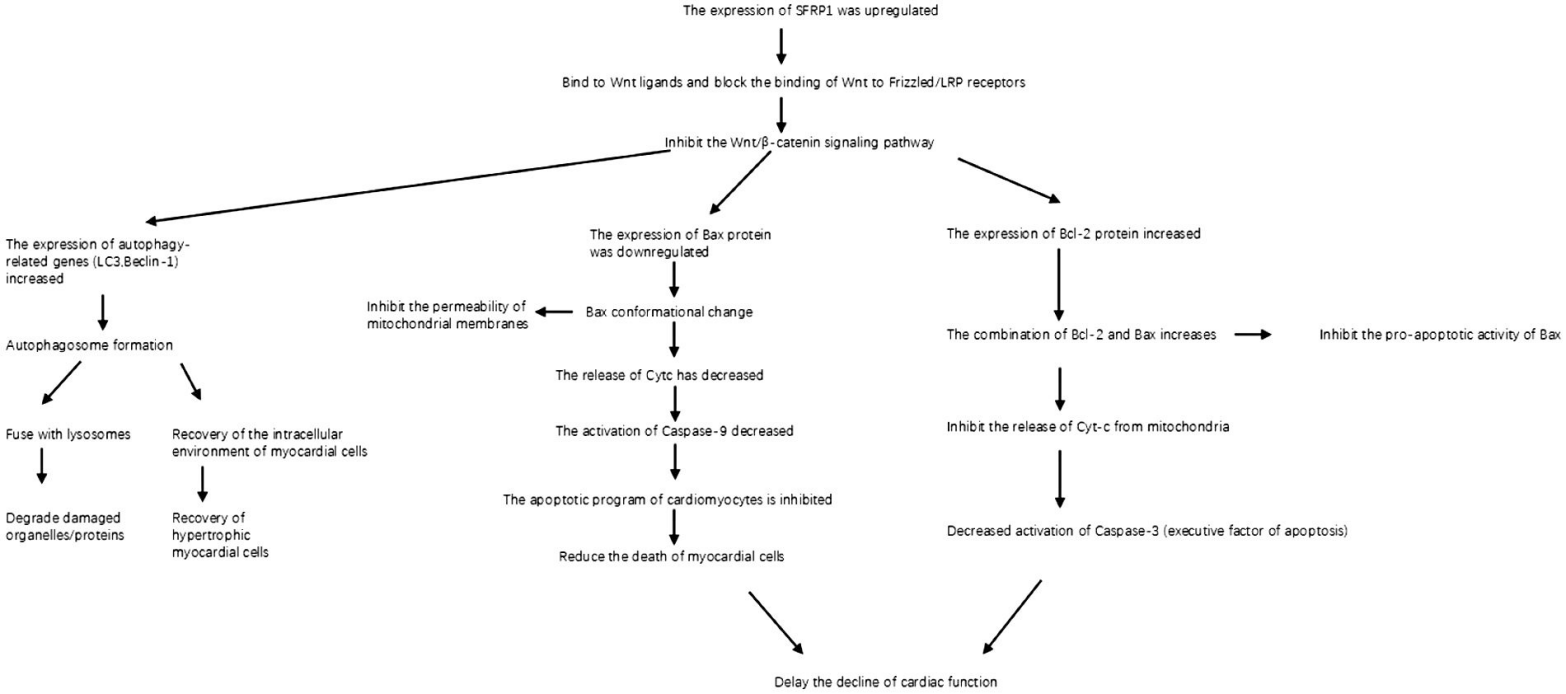
Sfrp1 initiates the autophagy program by inhibiting the Wnt/β-catenin signaling pathway, clears apoptotic cardiomyocytes, and regulates the mitochondrial apoptotic pathway. Flowchart depicting the effects of SFRP1 upregulation in cardiac cells. Key pathways include inhibition of the Wnt/β-catenin signaling, leading to downregulation of Bax protein, decreased Cyt-c release, and reduced Caspase-9 activation, thus inhibiting apoptosis and reducing myocardial cell death. Increased autophagy-related gene expression promotes recovery of myocardial cells and delays cardiac function decline.

This molecular evidences are revealed at the level of signal transduction. Sfrp1, by dual regulation of the Wnt pathway activity and the mitochondrial apoptosis pathway, constitutes a key molecular mechanism for inhibiting the programmed cell death of cardiomyocytes (63). Some scholars have, through their research, verified the efficacy of Sfrp1 in inhibiting cardiomyocyte apoptosis from multiple perspectives by detecting Bax, cytochrome (Cyt)c, cysteine aspartic acid-specific protease Caspase-3, and Bcl-2 (58, 78). Therefore, we can conclude that Sfrp1 can alleviate myocardial hypertrophy and delay HF by initiating the autophagy process in cells, eliminating apoptotic cardiomyocytes, and inhibiting cardiomyocyte apoptosis (Figure 6; Table 3).

**Table 3:** Summary of Key nodes and functional areas.

Functional link	The site of action of Sfrp1	Mode of action	Molecular mechanism
Wnt pathway inhibition	Cell membrane (bound to Wnt ligand)	Competitive inhibition of Wnt-frizzled/LRP binding	Block the nuclear translocation of β-catenin and inhibit transcriptional activity
Molecular mechanism	Cytoplasm, cell nucleus	Upregulate autophagy-related genes (LC3, Beclin-1)	Autophagosome formation and degradation, clearing damaged components
Regulation of Bax expression	Inner mitochondrial membrane, endoplasmic reticulum	Downregulation of the Bax gene at the transcriptional level	Reduce the permeability of the mitochondrial membrane and inhibit the release of Cyt c
Regulation of Bcl-2 Expression	Inner mitochondrial membrane, endoplasmic reticulum	Upregulation of the Bcl-2 gene at the transcriptional level	Antagonize the activity of Bax and maintain the stability of the mitochondrial membrane
Caspase-3 inhibition	cytoplasm	Reduce Cyt c release → inhibit Caspase-9/3 cascade activation	Upregulation of the Bcl-2 gene at the transcriptional level

#### 3.2.6 Sfrp1 synergistically inhibits CREB phosphorylation and restores calcium homeostasis in cardiomyocytes

Calcium metabolism disorder and abnormal CREB phosphorylation are mutually reinforcing pathological nodes in HF: Pathological calcium overload activates calcium-dependent kinases (e.g., CaMKII) to promote CREB phosphorylation, while hyperphosphorylated CREB further upregulates hypertrophy-related genes (e.g., ANP and β-MHC) and disrupts calcium-handling protein expression. Sfrp1 breaks this vicious cycle through two synergistic mechanisms: (1) directly inhibiting CREB phosphorylation to block calcium-dependent pathological transcription and (2) targeting key calcium-handling molecules to restore calcium homeostasis. This section systematically elaborates on this integrated regulatory process.

##### 3.2.6.1 Inhibition of CREB phosphorylation: Sfrp1 blocks dual activation pathways of CREB

CREB is a nuclear transcription factor that regulates myocardial hypertrophy by binding to CRE in target gene promoters. Its hyperphosphorylation in HF is mainly driven by two pathways:

1. Calcium-dependent pathway: Calcium overload activates the calcineurin-NFAT axis, which indirectly promotes CREB phosphorylation.

2. Wnt/β-catenin-dependent pathway: Hyperactivated Wnt/β-catenin upregulates upstream kinases of CREB (e.g., MAPK), enhancing its phosphorylation (58, 95). Sfrp1 inhibits CREB phosphorylation through dual targeting:

a. Competitive binding: Sfrp1 binds to the DNA-binding domain of CREB via its Frizzled-like domain, preventing CREB from recruiting co-activators (e.g., CBP/p300) and interacting with CRE, directly blocking its transcriptional activity (95, 96).

b. Pathway inhibition: By competitively binding Wnt ligands and suppressing Wnt/β-catenin activation, Sfrp1 reduces MAPK-mediated CREB phosphorylation and simultaneously alleviates calcium overload (detailed in Section 6.2) to inhibit the calcineurin-NFAT-CREB axis (82).

Experimental evidence: In AngII-induced cardiomyocyte hypertrophy models, exogenous Sfrp1 intervention reduced CREB phosphorylation (Ser133, the active site) by 42% compared with the control group and downregulated the expression of CREB target genes (ANP, β-MHC) by 35%–40%, confirming the inhibitory effect on CREB-mediated pathological transcription (52, 97).

##### 3.2.6.2 Restoration of calcium homeostasis: Sfrp1 targets sarcoplasmic reticulum and mitochondrial calcium handling

Abnormal calcium metabolism in HF mainly manifests as two defects: (1) SR calcium reuptake disorder (reduced SERCA2a expression) and (2) mitochondrial calcium overload (dysfunctional MCU) (97, 98). Sfrp1 restores calcium homeostasis by targeting these two defects, with no redundant regulatory steps:

1. Enhancing SR calcium reuptake: Sfrp1 inhibits Wnt/β-catenin signaling to upregulate SERCA2a expression. SERCA2a, the key SR calcium pump, accelerates the transport of cytoplasmic Ca^2+^ back to the SR, reducing diastolic cytoplasmic calcium concentration and alleviating myocardial relaxation dysfunction (97).

2. Preventing mitochondrial calcium overload: Sfrp1 upregulates MICU1 (a negative regulator of the MCU) by suppressing Wnt/β-catenin. MICU1 restricts excessive Ca^2+^ influx into mitochondria through MCU, avoiding mitochondrial membrane potential collapse and ATP depletion (99).

3. Blocking the calcium-dependent ROS-Ca^2+^ feedback loop: Mitochondrial calcium overload triggers excessive ROS production, which further damages calcium channels (e.g., RyR2 leakage). Sfrp1 reduces mitochondrial ROS generation by maintaining mitochondrial calcium homeostasis, thereby blocking this vicious cycle and protecting calcium-handling protein function. Blocking the calcium-dependent ROS-Ca^2+^ feedback loop: Mitochondrial calcium overload triggers excessive ROS production, which further damages calcium channels (e.g., RyR2 leakage). Sfrp1 reduces mitochondrial ROS generation by maintaining mitochondrial calcium homeostasis, thereby blocking this vicious cycle and protecting calcium-handling protein function (100). Experimental evidence: In Sfrp1-overexpressing mouse hearts, SERCA2a protein levels increased by 50%, and MICU1 expression was upregulated by 45% compared with HF models. Calcium transient detection showed that the time to 50% calcium decay (a marker of SR calcium reuptake) was shortened by 30%, confirming improved calcium handling (101, 102).

##### 3.2.6.3 Synergistic effect: how CREB inhibition and calcium regulation jointly alleviate myocardial damage

**Figure 7:**
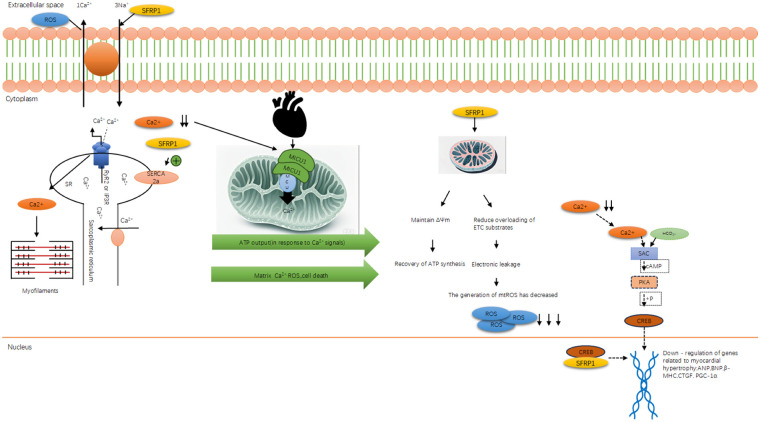
Sfrp1 synergistically regulates CREB phosphorylation and calcium metabolism. Diagram illustrating cellular signaling pathways involving calcium ions (Ca²+) and the role of SFRP1. It shows processes such as ATP synthesis, cell energy balance, and cellular responses. Key elements include a mitochondrial diagram highlighting MICU1, MCU, and the interactions affecting ATP output and calcium-induced cell death. Other paths detail electron transport chain activity and the involvement of SAC, cAMP, PKA, and CREB in gene regulation related to myocardial hypertrophy. The flow shows escalating reactions impacting cellular functions, modulated by SFRP1 and reactive oxygen species (ROS).

The regulatory effects of Sfrp1 on CREB phosphorylation and calcium metabolism are not independent but form a synergistic network:

1. Upstream: Sfrp1 inhibits Wnt/β-catenin to simultaneously achieve two effects: reducing CREB phosphorylation (blocking hypertrophy gene transcription) and upregulating SERCA2a/MICU1 (restoring calcium homeostasis) (103).
2. Downstream: Alleviated calcium overload further reduces the activation of calcium-dependent CREB kinases (e.g., CaMKII), forming a “calcium normalization → CREB inhibition enhancement”-positive feedback, which completely breaks the “calcium overload → CREB hyperphosphorylation → myocardial hypertrophy” vicious cycle in HF (104). This synergy ensures that Sfrp1 not only corrects the “final common pathway” of calcium metabolism disorder but also blocks the transcriptional drive of myocardial hypertrophy, achieving more comprehensive myocardial protection than single-target interventions (86, 105–107) (Figure 7; Table 4).

**Table 4:** Summary of key nodes and action sites of Sfrp1 inhibiting CREB phosphorylation to regulate calcium metabolism.

Regulatory target	Mode of action of Sfrp1	Site of action	Core effect
CREB	Competitive binding + Wnt/β-catenin inhibition	Nucleus	Reduces hypertrophy gene transcription
SERCA2a	Upregulation via Wnt/β-catenin suppression	Sarcoplasmic reticulum	Enhances cytoplasmic calcium reuptake
MICU1-MCU complex	Upregulation via Wnt/β-catenin suppression	Mitochondrial membrane	Prevents mitochondrial calcium overload
Mitochondrial ROS	Indirect inhibition via calcium normalization	Mitochondrial matrix	Blocks the ROS-Ca^2+^ feedback loop
