# Supplementary figures and images for: The mechanism of secreted frizzled-related protein 1 in alleviating cardiomyocyte injury and heart failure
Source: Front Cardiovasc Med. 2026 Jan 16;12:1676224. doi: 10.3389/fcvm.2025.1676224 (PMC12855572; doi:10.3389/fcvm.2025.1676224)

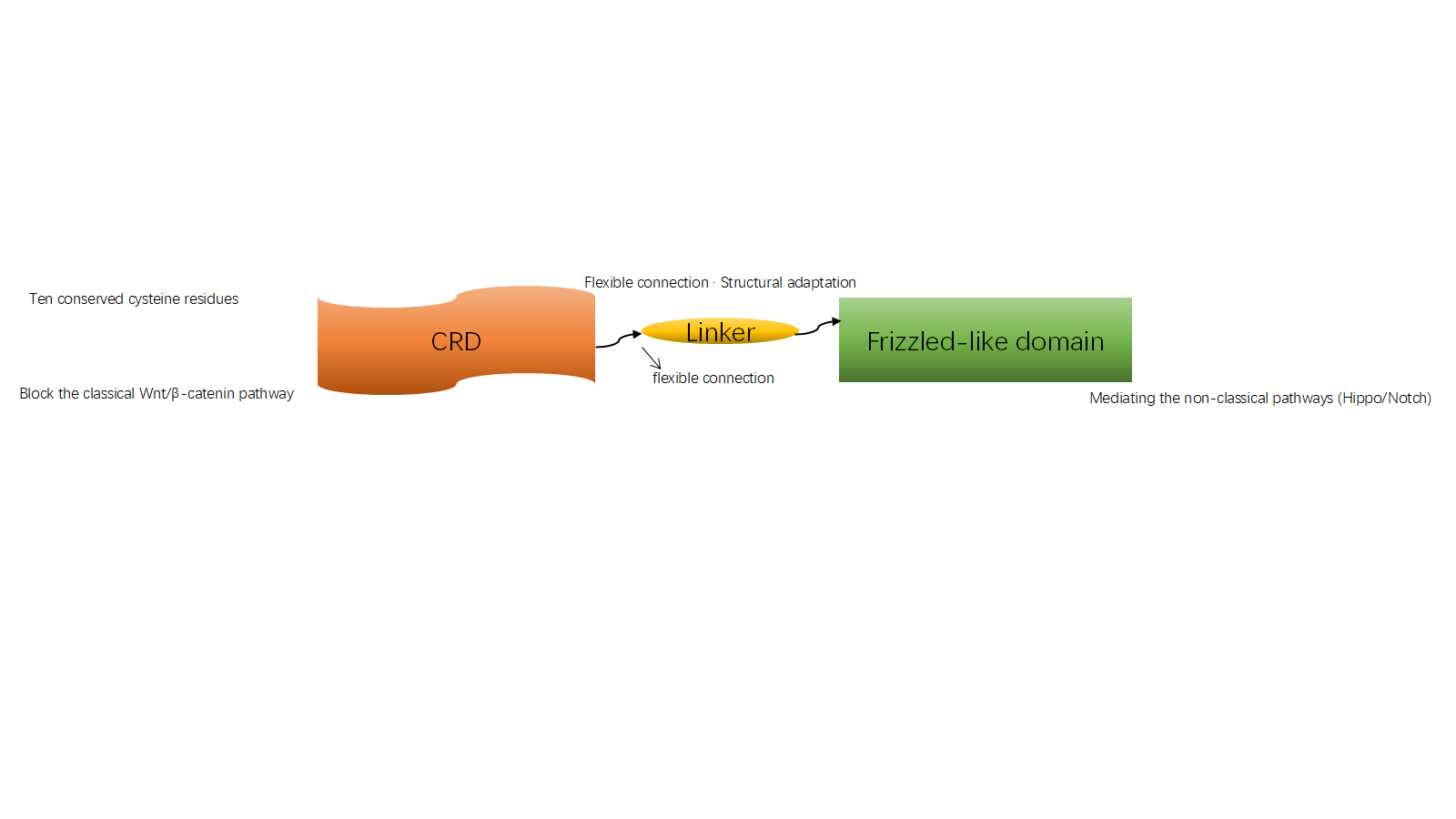

Supplement: Supplementary file 1 [file Image1.png]

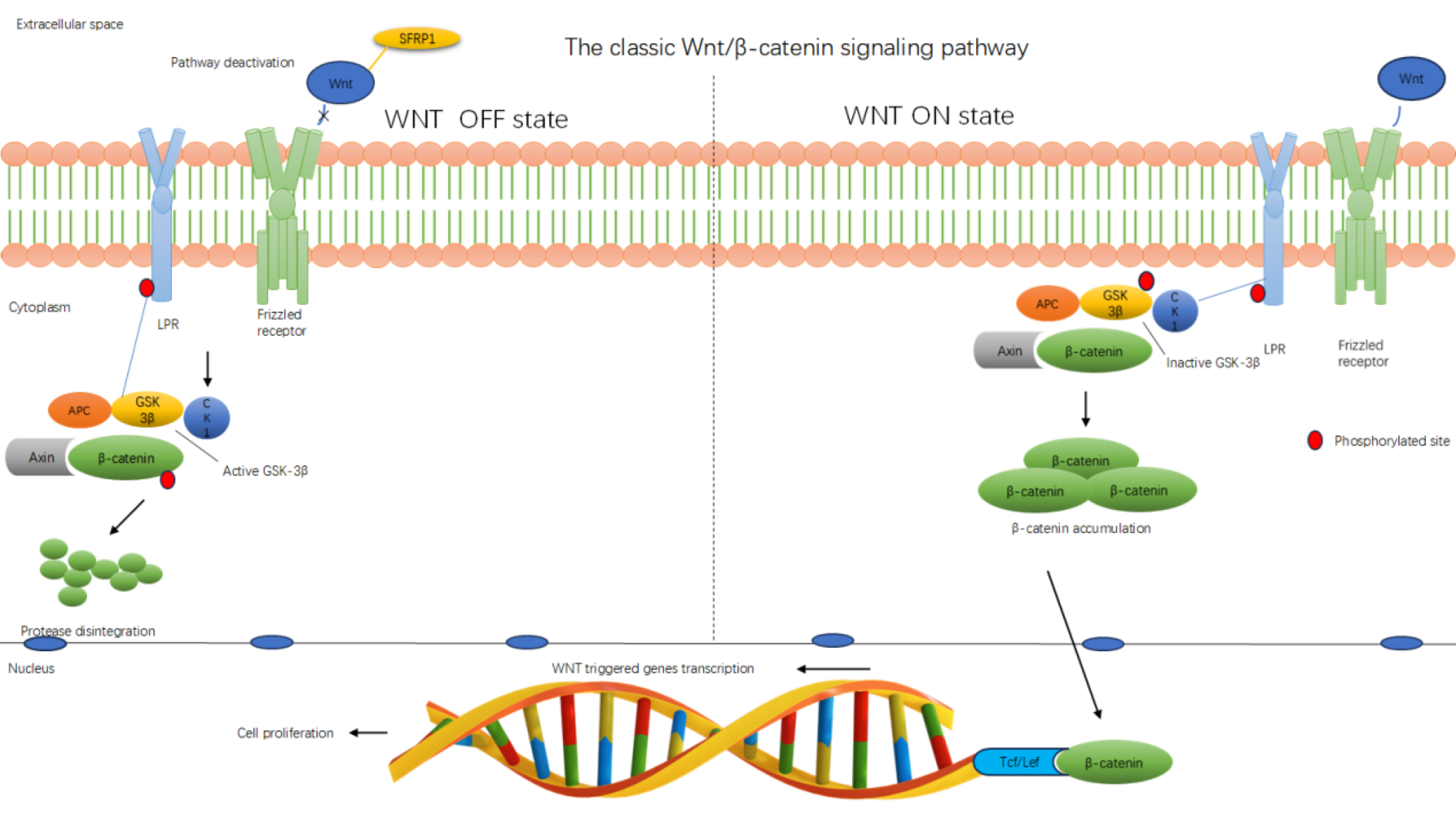

Supplement: Supplementary file 2 [file Image2.png]

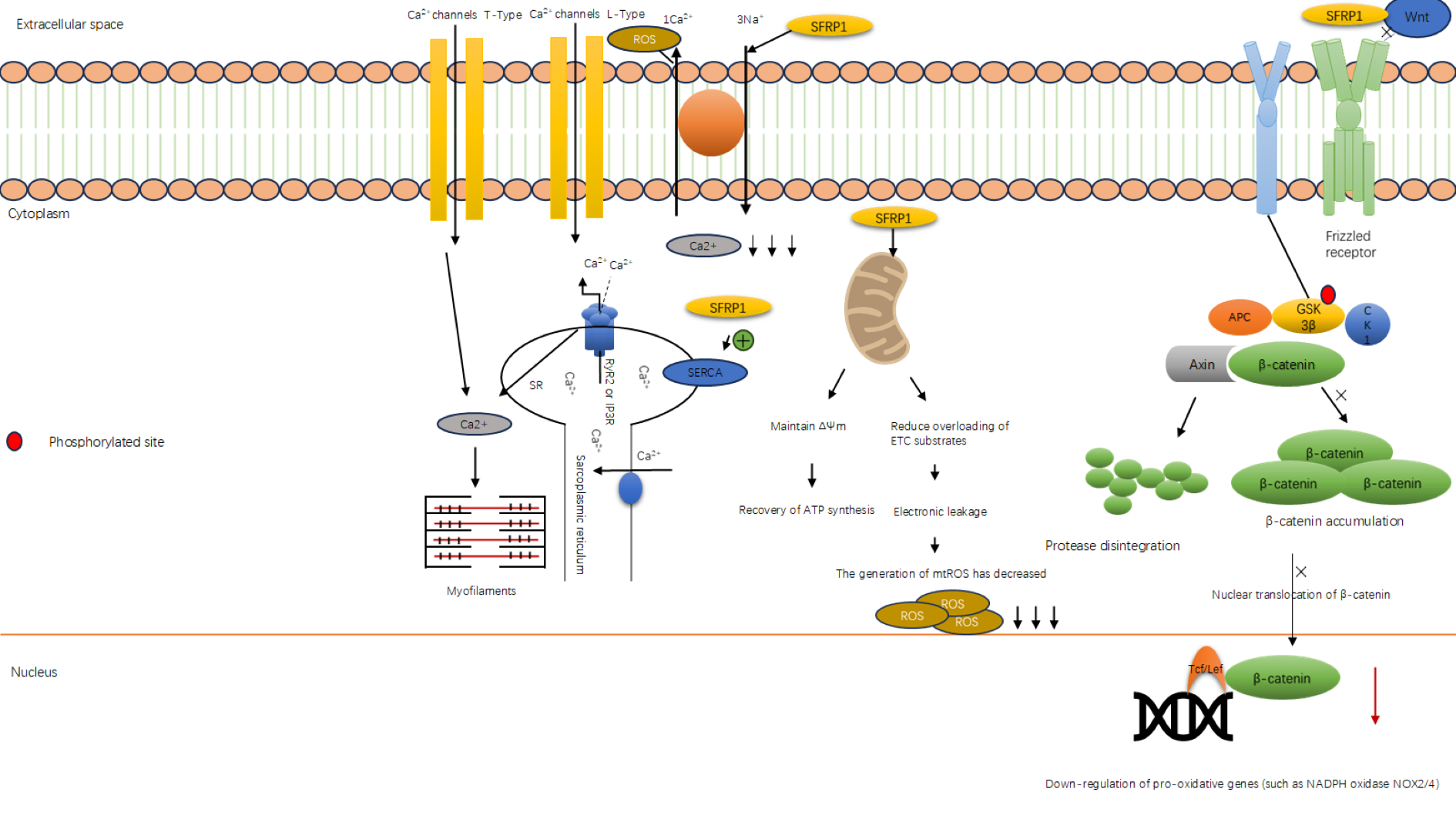

Supplement: Supplementary file 3 [file Image3.png]

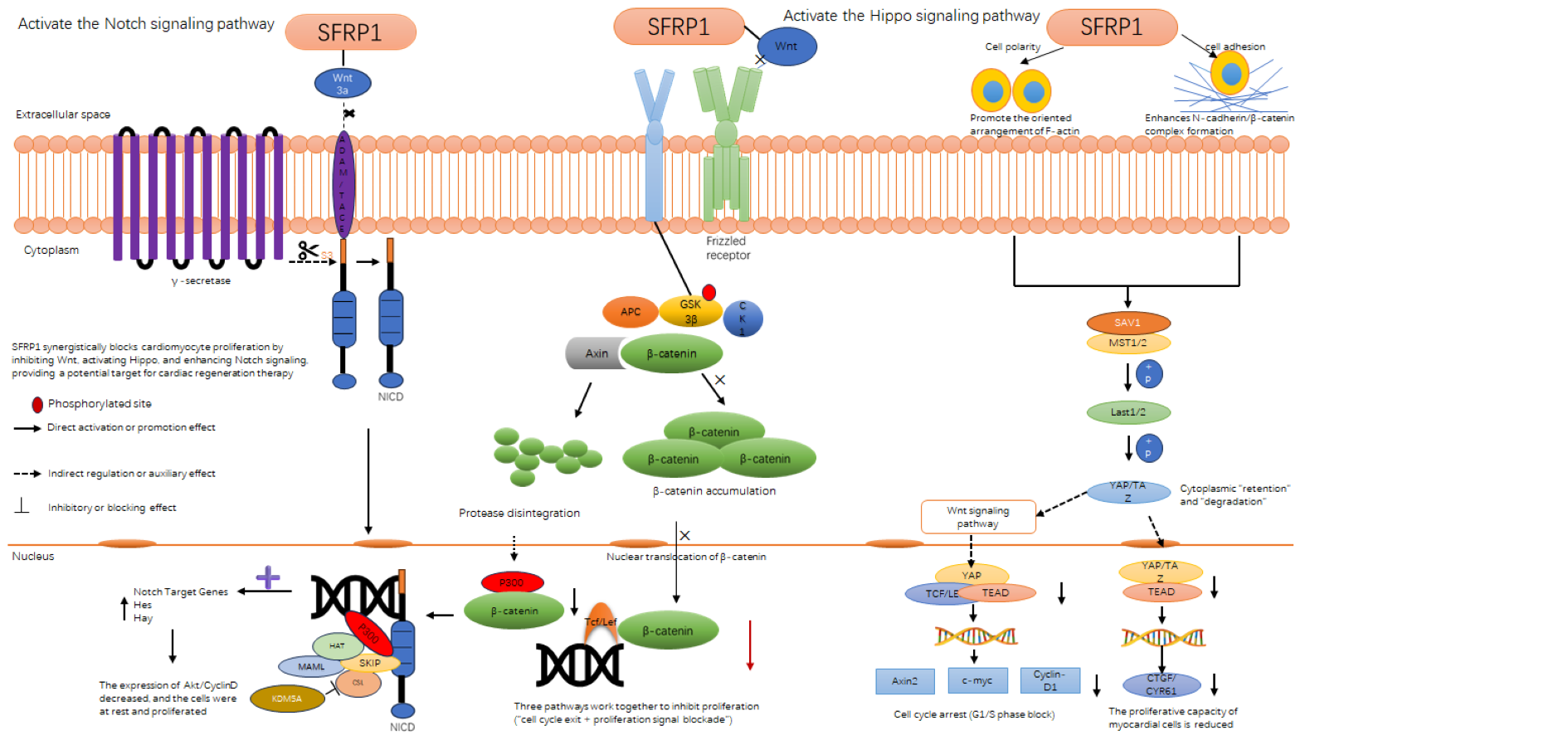

Supplement: Supplementary file 4 [file Image4.png]

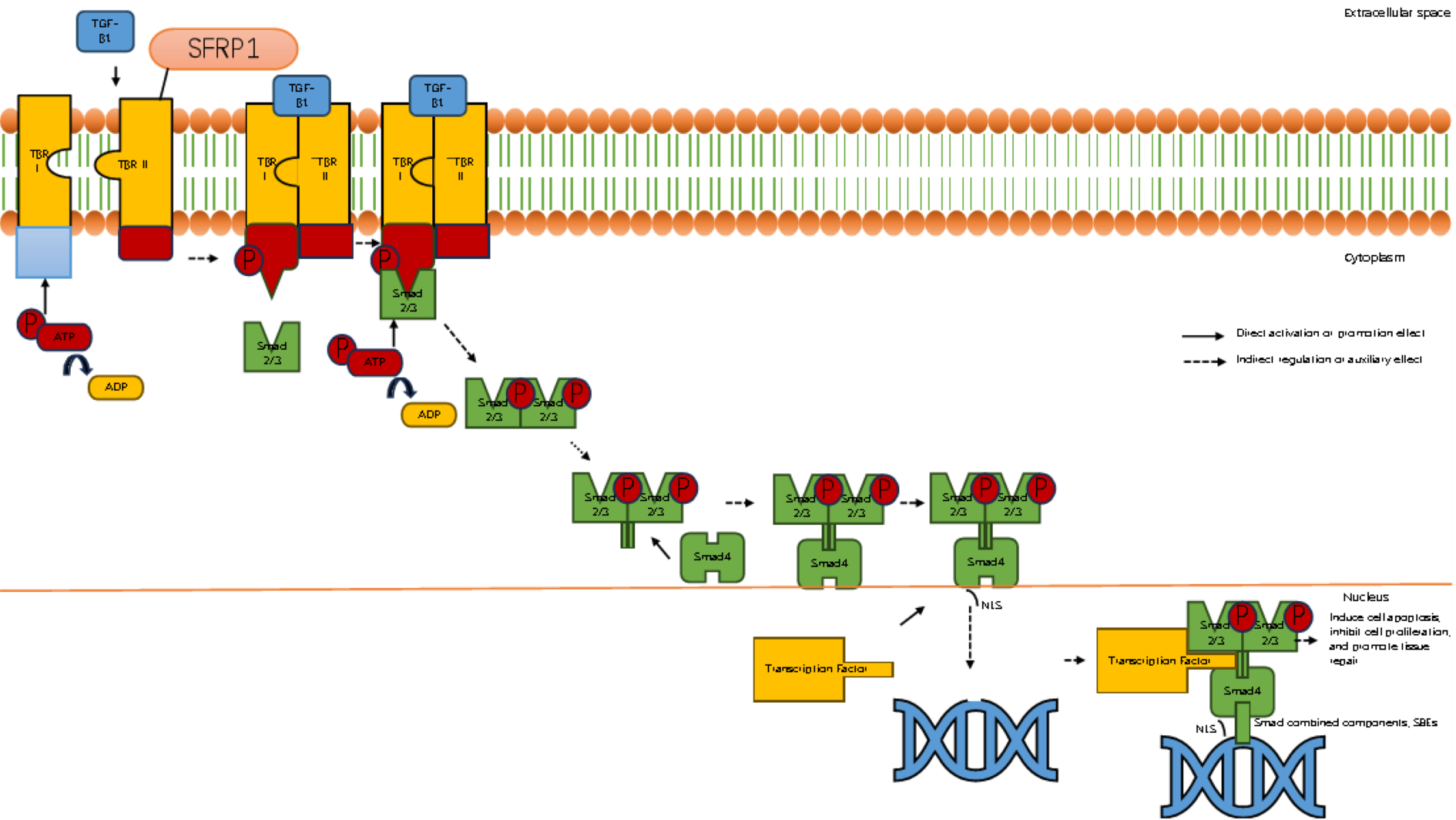

Supplement: Supplementary file 5 [file Image5.png]

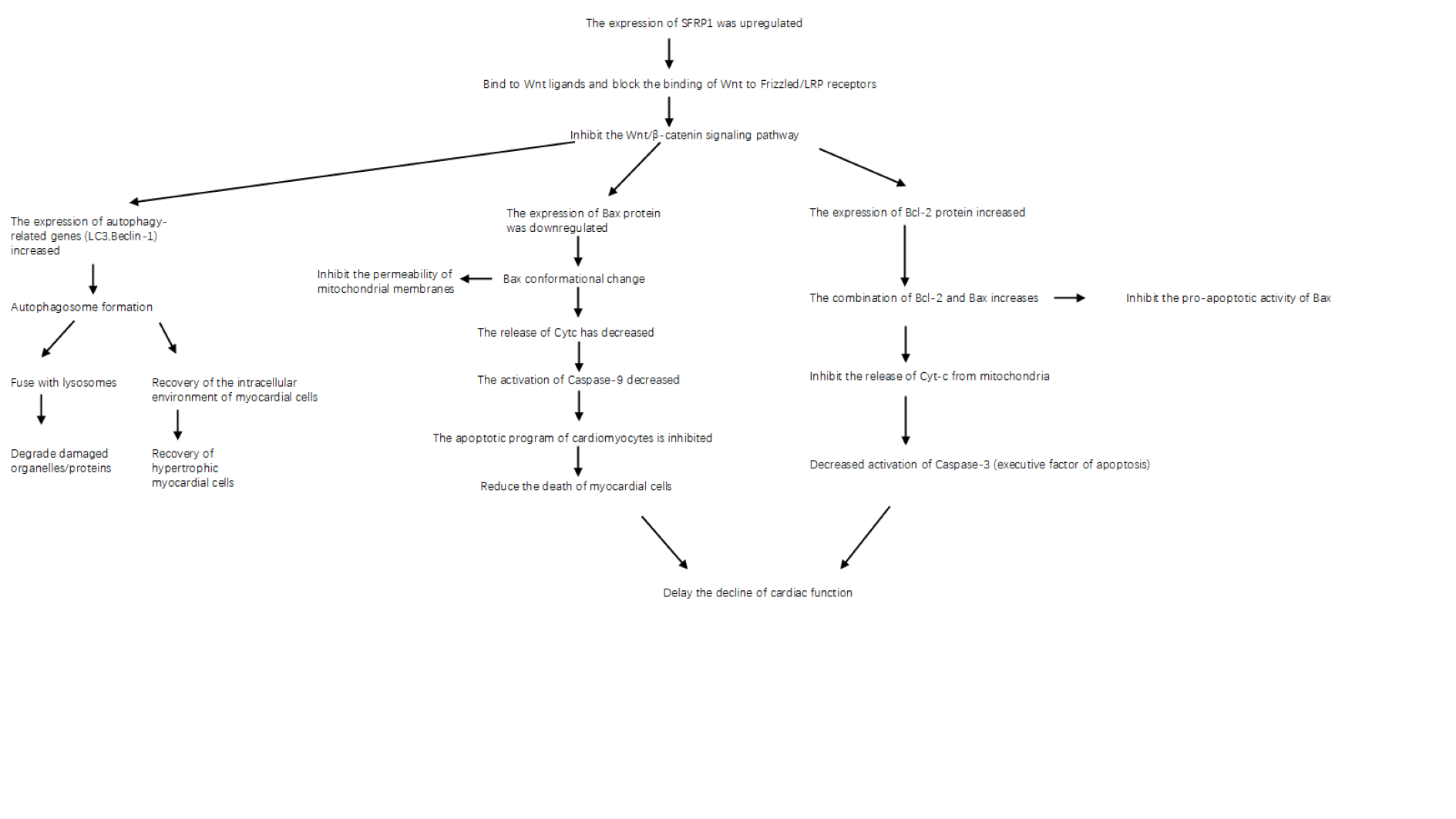

Supplement: Supplementary file 6 [file Image6.png]

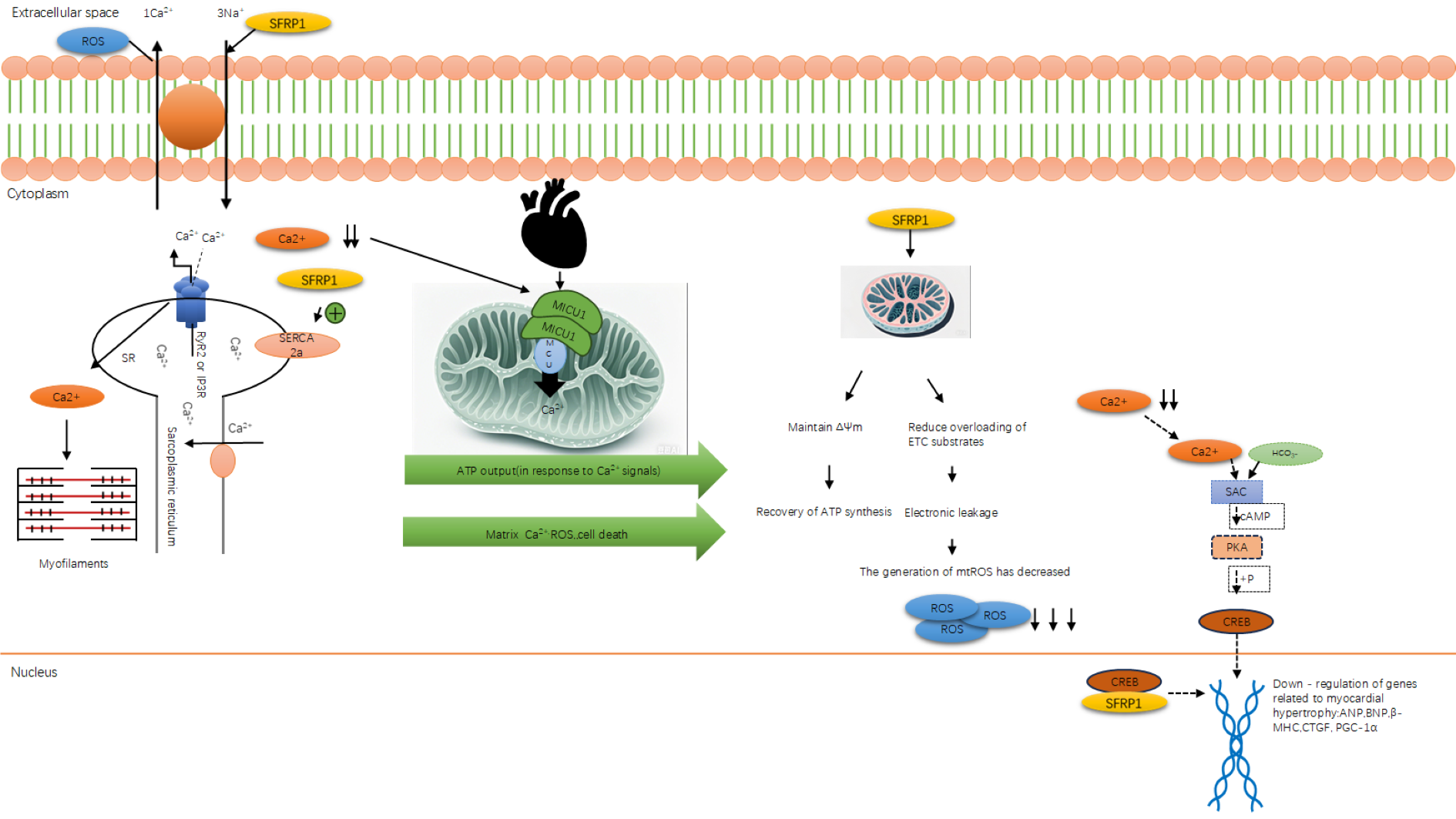

Supplement: Supplementary file 7 [file Image7.png]
